# How to Reproduce in the Siberian Winter: Proteome Dynamics Reveals the Timing of Reproduction‐Related Processes in an Amphipod Species Endemic to Lake Baikal

**DOI:** 10.1002/ece3.71675

**Published:** 2025-07-23

**Authors:** Polina Lipaeva, Polina Drozdova, Kseniya Vereshchagina, Lena Jakob, Kristin Schubert, Daria Bedulina, Till Luckenbach

**Affiliations:** ^1^ Department of Ecotoxicology Helmholtz Centre for Environmental Research—UFZ Leipzig Germany; ^2^ Institute of Biology Irkutsk State University Irkutsk Russia; ^3^ Alfred Wegener Institute Helmholtz Centre for Polar and Marine Research Bremerhaven Germany; ^4^ Department of Molecular Toxicology Helmholtz Centre for Environmental Research—UFZ Leipzig Germany

**Keywords:** cold adaptation, endemic amphipod, Lake Baikal, proteome dynamics, reproduction strategy

## Abstract

The winters in the region of the vast global freshwater biodiversity hotspot Lake Baikal are extremely cold. Although the conditions for reproduction may seem unfavorable during winter, the lake is inhabited by a major endemic winter‐reproducing amphipod species complex. Compared with Baikal's summer‐reproducing amphipod species, the duration of a reproduction cycle in the winter‐reproducing species is more extended. We hence hypothesized that in those species, reproduction‐related processes dependent on external resources are scheduled to occur outside of winter when the conditions are more advantageous. To receive insights into the ongoing processes, we analyzed sex‐specific seasonal proteome dynamics in *Eulimnogammarus verrucosus* as a representative of the winter‐reproducing amphipod species. Individuals of the species were collected during five field samplings from the beginning of fall to the following summer (2019/2020) and their proteomes were analyzed. Especially, the female proteomes were dominated by sampling time point‐specific hallmarks of reproduction‐related processes and events. It was evident that the formation of the oocytes in female *E. verrucosus* already took place in the summer. Embryo development, not depending on external resources but fueled by the yolk reserves in the egg, proceeded over the winter, and juveniles hatched from the eggs in the following spring. Adjustments of the amphipods of both sexes to environmental winter conditions were reflected by abundance changes of digestive system‐related enzymes, indicating a proteome response to seasonal diet changes, and of enzymes involved in RNA biosynthesis, protein folding, and homeoviscous adaptation processes, possibly related to decreasing water temperatures. The characteristics of the proteome dynamics revealed here, set in relation to season‐specific environmental parameters, indicate a strategy of a cold‐adapted amphipod to cope with the unique and extreme environmental conditions of Baikal, which is directed to the pace and timing of the resource‐dependent reproduction‐related processes.

## Graphical abstract

The vast Lake Baikal in Siberia is located in a region with a continental climate, implying pronounced seasonal temperature differences. Remarkably, reproducing during the extremely cold winters when the lake is covered by a thick ice layer is a common trait in Baikal's endemic littoral amphipods. By melding animal‐specific molecular and environmental data our study illustrates the strategy enabling an endemic amphipod species, studied exemplary for the endemic winter‐reproducing amphipod species complex, to utilize the harsh conditions in Baikal in winter for reproduction. As this species complex is dominant in Baikal's littoral amphipod fauna, the here revealed strategy of timing of reproduction‐related events in the animals appears to have evolved as an advantageous, competitive key feature in Baikal's specific environmental conditions.

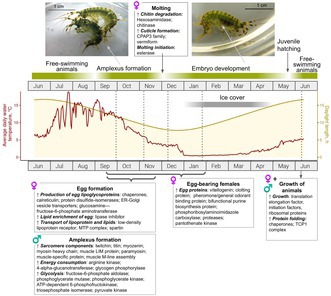

## 1 Introduction

**FIGURE A1:**
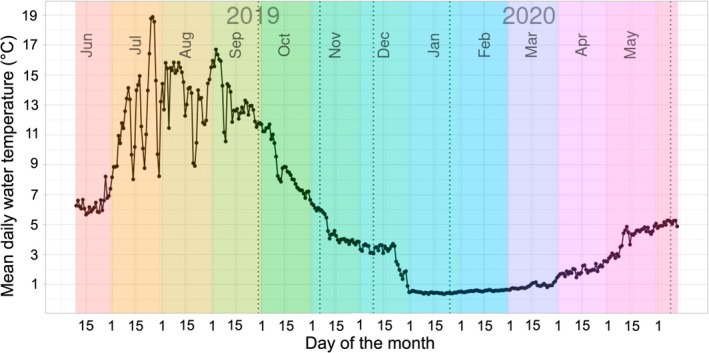
(Supplementary to Figure 6) Water temperature profile in Lake Baikal close to the sampling location for *E. verrucosus* . The dots represent mean daily water temperatures calculated from temperature recordings performed every 3 h. For better visualization, consecutive dots were connected. The vertical dotted lines indicate the time points of the amphipod samplings (September 2019–June 2020).

Lake Baikal, the world's oldest, by volume largest and deepest lake, is located in Eastern Siberia, a region with an extreme continental climate. The −20°C isotherm transects Lake Baikal from December until February, and in winter, the lake is covered by ice with a thickness of up to 1 m (Shimaraev and Troitskaya 2018; Todd and Mackay 2003). In summer, the water temperature close to shore can rise to around 20°C (Fedotov and Khanaev 2023; see also Figure A1 in the Appendix); the mean surface water temperature from May to September is close to 10°C (Shimaraev and Troitskaya 2018). Baikal's water is classified as ultra‐oligotrophic and has exceptionally high oxygen levels in all water depths (Khodzher et al. 2017).

UNESCO world heritage site Lake Baikal is a global biodiversity hotspot, with more than half of the animal species inhabiting the lake being endemic (Timoshkin 2001). Baikal's amphipods, all of them endemic, equal approximately 20% of all so far described freshwater amphipod species (Väinölä et al. 2008). Over 350 amphipod species and subspecies comprise approximately 90% of Baikal's benthic biomass (Takhteev 2019).

**FIGURE A2:**
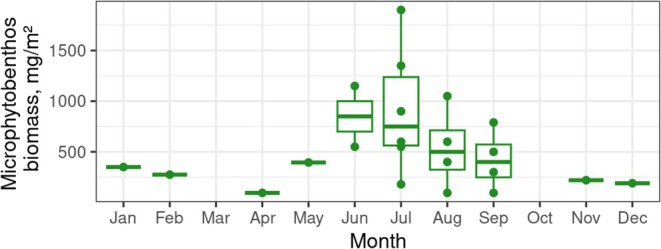
(Supplementray to the section “Timing of reproduction‐related processes and environmental parameters”) Abundance dynamics of microphytobenthos in the Lake Baikal littoral over the year. Microphytobenthos abundance is expressed as biomass given for different months. The graph is based on data published earlier (Pomazkina et al. 2008), which were retrieved over several years (1997–2002). The investigated transect close to the village of Bolshie Koty, where the *E. verrucosus* sampling site for this study is located, is presumably *E. verrucosus* habitat.

Lake Baikal's amphipod species are cold‐adapted, maintaining high physiological activity in the low energy conditions at water temperatures close to 0°C in winter (Lipaeva et al. 2021; Vereshchagina et al. 2021). There are indications that the species' high level of activity at low water temperatures is enabled by enhanced mitochondrial functioning and the species' ability to maintain cell membrane fluidity in cold conditions (Lipaeva et al. 2021; Vereshchagina et al. 2021). Furthermore, the specific environmental conditions of Baikal in winter, such as a vast body of permanently liquid and highly oxygenated water underneath a thick ice cover, enable high mobility and metabolic activity of the amphipods (Kozhov 1963). Additionally, food resources, such as algae, are available in winter, although to a lesser extent than in summer: High algal growth in spring beneath the ice cover and during summer is indicated by peaks of primary production, which proceeds also in the other seasons—including winter—but at a lower rate (Hampton et al. 2008). A similar trend as for the pelagic algae was also observed for the season‐dependent abundance of microphytobenthos in the littoral, which we used here as a proxy for season‐dependent food abundance for the littoral amphipod species studied here, *Eulimnogammarus verrucosus*: The biomass of microphytobenthos measured in the summer months tended to be higher than in winter [(Pomazkina et al. 2008); see below and Figure A2 in the Appendix].

Amphipod species complexes of Baikal are distinguished by their reproduction times, that is, summer, winter, or throughout the year (Bazikalova 1941). Most species inhabiting the littoral zone, including numerous *Eulimnogammarus* species, belong to the winter‐reproducing species complex (Gavrilov 1949).

Reproduction requires increased availability of energy resources, which raises the question of how winter reproduction can be such a common trait in Baikal amphipods, given that nutritive primary producers are less abundant in winter than in summer (Hampton et al. 2008; Kozhova and Izmest'eva 1998; Pomazkina et al. 2008; Popova et al. 2012). Moreover, the water temperatures are below the species' preferred temperatures (Timofeyev et al. 2001), which probably are the optimum temperatures for metabolism. Whereas the reproduction cycle of Baikal's littoral summer‐reproducing amphipods is comparatively short, with two offspring cohorts per season, Baikal's winter‐reproducing amphipods are characterized by a univoltine annual life cycle with an extended reproduction cycle (according to the classification of life histories of gammaridean amphipods by Wildish 1982). Within this life cycle, egg laying, copulation, embryo development, and hatching occur in the coldest months of the year, from fall until the following summer, a pattern also observed for the species investigated here (Bazikalova 1941; Takhteev 2000). The extended reproduction cycle of the winter‐reproducing amphipod species raises the question of whether indeed all reproduction‐related processes take place during winter. We thus hypothesized that certain resource‐dependent processes take place outside the winter when conditions are less harsh.

To test this hypothesis, we investigated time‐of‐the‐year‐dependent proteomic profiles in the amphipod *E. verrucosus* (Gavrilov, 1949), exemplarily for the winter‐reproducing amphipod species from Lake Baikal. Specimens of this species were sampled in the field over several months from the beginning of fall until the following summer in two consecutive years (2019/2020). We chose to perform proteomic analyses of the sampled amphipods, as proteomics has already proven powerful in earlier studies to analyze cellular and physiological processes related to thermal adaptation in amphipods from Lake Baikal, nonmodel species without available genome sequences (Bedulina et al. 2021; Lipaeva et al. 2023). Data were examined for the occurrences of sex‐specific reproduction‐related processes and of metabolic adjustments to environmental conditions; those were aligned with the times of season‐specific environmental (temperature, ice cover, daylight length, food abundance) and reproduction‐related parameters (times of mating, embryo growth, and release of juveniles by females). To be able to determine the sex specificity of the analyzed individuals' proteomic patterns, the sex of the amphipods was identified macroscopically for individuals that were caught as a mating pair or based on the abundance levels of certain vitellogenin (Vg) isoforms used as molecular sex markers (see below and Text S2 in the Appendix).

## 2 Materials and Methods

### 2.1 Studied Species: Description and Characteristics

The here‐studied *E. verrucous* (Gerstfeldt, 1858) is an omnivorous amphipod species with a lifespan of around 5 years (Govorukhina 2005). It dominates the upper and mid‐rocky littoral zone (water depths 0–15 m) of Lake Baikal (Bazikalova 1945; Kravtsova et al. 2004). The species is quite conspicuous because of its comparatively large body size [adults ~30 mm (Timofeyev et al. 2001)] and its coloring (see photographs of *E. verrucosus* in Figure 6). *Eulimnogammarus verrucous* is stenothermic and migrates to deeper, cooler waters when the temperatures in the shallow water close to Baikal's shore rise in the summer (Jakob et al. 2016; Weinberg and Kamaltynov 1998).

The species' three geographically separate genetic lineages inhabit different regions of Baikal (Gurkov et al. 2019). The sampling site of the here examined *E. verrucosus* is located in the region occupied by the species' western (“W”) genetic lineage (Drozdova et al. 2022; Gurkov et al. 2019).

**FIGURE A3:**
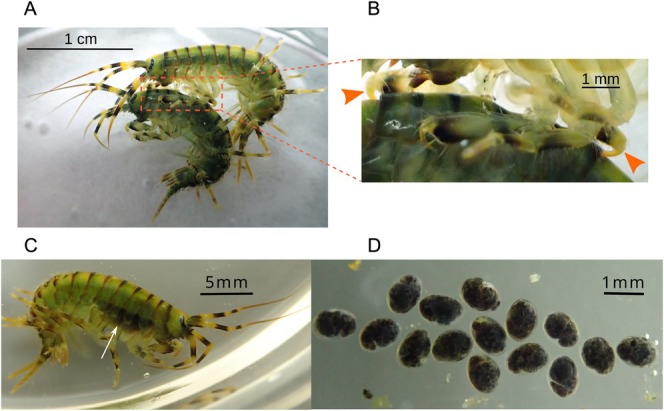
(Supplementary to Figure 1) Photographs of *E. verrucosus* during the mating/reproductive period. (A) Male (above) and female *E. verrucosus* forming an amplexus. (B) Body parts, with which the male holds on to the female *E. verrucosus* when in amplexus. Orange arrows mark two dactyls of male extremities that are used to grasp the female between the head and the first segment and between the fifth and sixth segments. (C) Female *E. verrucosus* with eggs in the brooding chamber (white arrow); (D) eggs washed out from a female *E. verrucosus* individual sampled in January.

Reproduction of *E. verrucosus* takes place in the winter at water temperatures of 0°C–6°C (Gavrilov 1949). A laboratory experiment indicated that exposure of *E. verrucosus* to a water temperature within this range (1.5°C) triggers the upregulation of genes associated with reproduction‐related processes in this species (Lipaeva et al. 2021). Individuals start occurring in amplexus (precopulatory mate guarding, see photos in Figure 6 and Figure A3A in the Appendix) in September ([Bazikalova 1941]; own observations), remaining in this state for 1–2 months. *Eulimnogammarus verrucosus* females start to become ovigerous in the second half of October at temperatures around 6°C, and descendants are released as juveniles from the females' marsupia from the beginning of May (Bazikalova 1941; A.Y. Bazikalova 1945; Bekman and Den'gina 1969; Gavrilov 1949). Embryo development of *E. verrucosus* lasts about 6–7 months; a comparatively extended embryo development is typical for Baikal's winter‐reproducing amphipod species complex.

*Eulimnogammarus verrucous* is highly abundant in Lake Baikal's littoral zone; it is not endangered or protected, and no permission to collect the species was required.

### 2.2 Field Samplings

Adult *E. verrucosus* individuals (> 15 mm) were caught with a hand‐net by kick‐sampling near the Biological Station of Irkutsk State University in Bolshie Koty (51°54′11.67″ N, 105°4′7.61″ E) at 0.5–1.5 m water depths. Amphipods in mating pairs were caught in September and November 2019; in the other months, adult *E. verrucosus* were collected as single individuals (refer to Table 1 for details). For proteomics analyses, animals were flash‐frozen in liquid nitrogen in the field and stored at −80°C until further analysis.

**TABLE 1:** Details on *E. verrucosus* field samplings including the following information: Sampling month and date; water temperature at the sampling site; ice coverage (yes/no); the number of sampled *E. verrucosus* individuals of which the proteomes were analyzed; sampled animals being in amplexus (yes/no); and numbers of sampled females (f)/males (m).

Month	Date	Water temperature, °C	Ice	Numbers of individuals	In amplexus	Sex
September	2019‐09‐29	10.5	No	10	Yes	5 f/5 m^a^
November	2019‐11‐06	6.4	No	10	Yes	5 f/5 m^a^
December	2019‐12‐09	2.3	No	28	No	15 f/13 m^b^
January	2020‐01‐25	0.3–0.4	Yes	5	No	4 f/1 m^b^
June	2020‐06‐09	~7	No	11	No	ND^c^

*Note:* Prior to sampling in January 2020, ice covering the water at the sampling site had to be removed. Upon sampling, *E. verrucosus* individuals at the given numbers were frozen immediately in liquid N_2_ in the field for proteome analysis. The sex of each animal was indicated by its position in an amplexus (Figure A3A in the Appendix; September and November samplings) or based on the here identified molecular sex markers for the animals sampled in December and January (Figure 1 and Text S2 in the Appendix).

^a^ Sex indicated by an individual's position in an amplexus.

^b^ Sex indicated by molecular sex markers (Text S2 in the Appendix).

^c^ The animals' sex could not be determined (ND).

### 2.3 Acquisition and Processing of Proteomics Data

Protein extraction from complete frozen animals, preparation of the extracts for LC–MS/MS, and LC–MS/MS were performed as described in Lipaeva et al. (2023) and in Text S1 in the Appendix. The obtained MS raw data were processed using MaxQuant v1.6.17.0 with isobaric matching between runs and peptide‐spectrum match level normalization (Yu et al. 2020). Peptide searching was performed against a customized transcriptome database with data from amphipod species *E. verrucosus* , *Eulimnogammarus cyaneus*, and *Gammarus lacustris* . The transcriptome database was built with de novo assembled raw mRNA sequencing data from BioProject PRJNA660769 using rnaSPAdes (Bushmanova et al. 2019). Corrected reporter intensities were extracted from a proteinGroup text file generated by MaxQuant. The analysis of intensity values was performed with in‐house scripts (https://github.com/PolinaLip/SeasonalExp_Proteome_BaikalAmphipods). Sample loading normalization was done by scaling each sample's total reporter ion intensity to the average total intensity across all samples (Plubell et al. 2017).

### 2.4 Molecular Sex Markers and Principal Component Analysis

**FIGURE A4:**
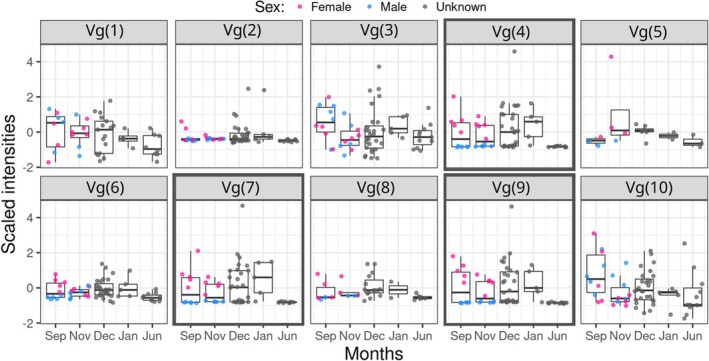
(Supplementary to Figure 1) Abundance levels of different vitellogenin (Vg) isoforms in the proteomes of *E. verrucosus* from samplings in the different months. The graphs with bold frames show the abundance levels of the Vg isoforms used as molecular sex markers in this study [Vg(4), Vg(7), Vg(9)]. Those Vg isoforms with clear differences in abundance in *E. verrucosus* females and males were identified in individuals with the sex known based on the position in the amplexus (September and November samplings; Figure A3).

**FIGURE A5:**
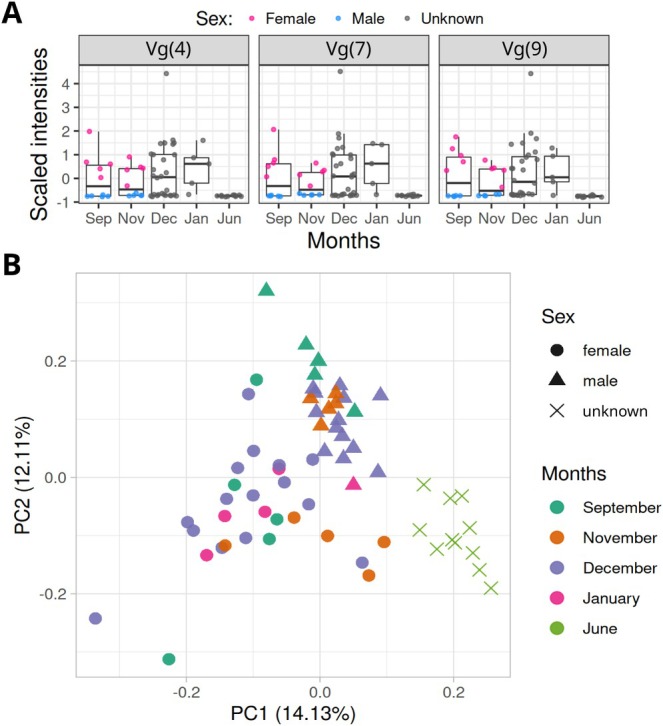
(Supplementary to Figure 1) (A) Relative abundance levels of the three vitellogenin (Vg) isoforms used as sex markers in the proteomes of *E. verrucosus* sampled in the field. Information on the sex of the analyzed animals came from an individual's position in the amplexus (September and November samplings; Figure A3) or from the abundance levels of the respective Vg isoforms (December and January samplings; no clear sex indication for *E. verrucosus* individuals sampled in June). (B) Results of a PCA on the abundance data for all proteins in the proteomes of the analyzed *E. verrucosus* individuals. Information on the sex of each analyzed individual (abundance level of the corresponding Vg isoform protein level/position in an amplexus) and the respective sampling month has been included.

Vitellogenin (Vg), the egg yolk precursor protein, was earlier found to show substantially higher abundance levels in females than in males of an amphipod species and of other crustaceans [(Jubeaux et al. 2012) and references therein]. Based on these findings, Vg levels were selected here as a sex marker for *E. verrucosus* . The sex of each examined *E. verrucosus* individual was determined by using a support vector machine (SVM) (R package e1071 version 1.7–9) that was trained with the abundance level data for three Vg isoforms (Figures A4 and A5 in the Appendix) in the proteomes of *E. verrucosus* individuals sampled as mating pairs and with their sex therefore known (see also Text S2 in the Appendix for details).

Principal component analysis (PCA) of the abundances of all proteins in the proteomes of field‐sampled *E. verrucosus* was performed with the prcomp function from stats package version 4.0.3 in R.

### 2.5 Weighted Gene Correlation Network Analysis (WGCNA)

The whole‐proteome data were analyzed by weighted gene correlation network analysis (WGCNA), a *p* value‐independent co‐expression network approach. The network was constructed across all measured samples by R package WGCNA (Langfelder and Horvath 2008), version 1.70.3. For WGCNA, the soft power threshold was set to 5. The Topology Overlap Matrix (TOM) was created using a minimum module size (minModuleSize parameter) of 25 and module detection sensitivity (deepSplit parameter) of 4. For each of the obtained modules, significantly enriched gene ontology (GO) terms were determined using R package topGO (Alexa et al. 2006), version 2.42.0. WGCNA module membership values were scaled independently by the highest membership value for each module. Then, proteins with scaled module membership values over 0.75 were taken for the enrichment analysis.

### 2.6 Acquisition of Ecological Parameter Data (Temperature, Daylight Length, Ice Cover, and Food Abundance)

The water temperature of Lake Baikal close to the *E. verrucosus* sampling site was monitored during the study period using a data logger (no. DS1922L, iButton, Maxim Integrated, CA, U.S.), mounted on a wooden pillar at a water depth of 3 m. The pillar is part of a pier (“Scuba diver's pier”) at the Lake Baikal shore close to the village of Bolshie Koty, South‐West Baikal (51°54′11.67″ N, 105°4′7.61″ E). The temperature was logged once every 3 h. Based on this data, the average daily values were calculated and plotted (see Figure A1 in the Appendix).

Daylight lengths for the sampling site coordinates were calculated with the getSunlightTimes function from the suncalc package for R (https://CRAN.R‐project.org/package=suncalc).

The duration of ice cover on Lake Baikal at the sampling site within our sampling time period was determined using the EOSDIS Worldview database satellite images of Southern Baikal (https://worldview.earthdata.nasa.gov/?v=103.38055265608253,50.996771796999816,106.16096234033694,52.17844591280794&t=2020‐01‐01‐T22%3A38%3A36Z). The ice cover started to form at the beginning of January 2020, was established by January 15th, and persisted until mid‐April; by May 1st the water was ice‐free.

Published data for microphytobenthos abundance (Pomazkina et al. 2008) were used as a proxy for the season‐characteristic food abundance for *E. verrucosus* in the lake. The microphytobenthos abundance data were collected near our *E. verrucosus* sampling site in Lake Baikal and are expressed as biomass (in mg) per m^2^ (see Figure A2 in the Appendix).

## 3 Results

To reveal the seasonal physiological and reproduction‐related processes in *E. verrucosus* from Lake Baikal, 5–28 specimens of this species were collected in the field in different months and seasons (September, November, December 2019; January, June 2020; details in Table 1) and their proteomes were analyzed by LC–MS/MS (Materials and Methods and Text S1 in the Appendix). Upon data processing, the total number of identified proteins was 1431. The *E. verrucosus* proteomes were analyzed for trait‐specific patterns. The sex of mating amphipods was identified by their position in the amplexus (Figure 6 and Figure A3A in the Appendix); the sex of nonmating individuals was determined based on the abundances of Vg protein isoforms, used as molecular sex markers (see below and Text S2 in the Appendix).

### 3.1 Molecular Sex Markers

**FIGURE 1:**
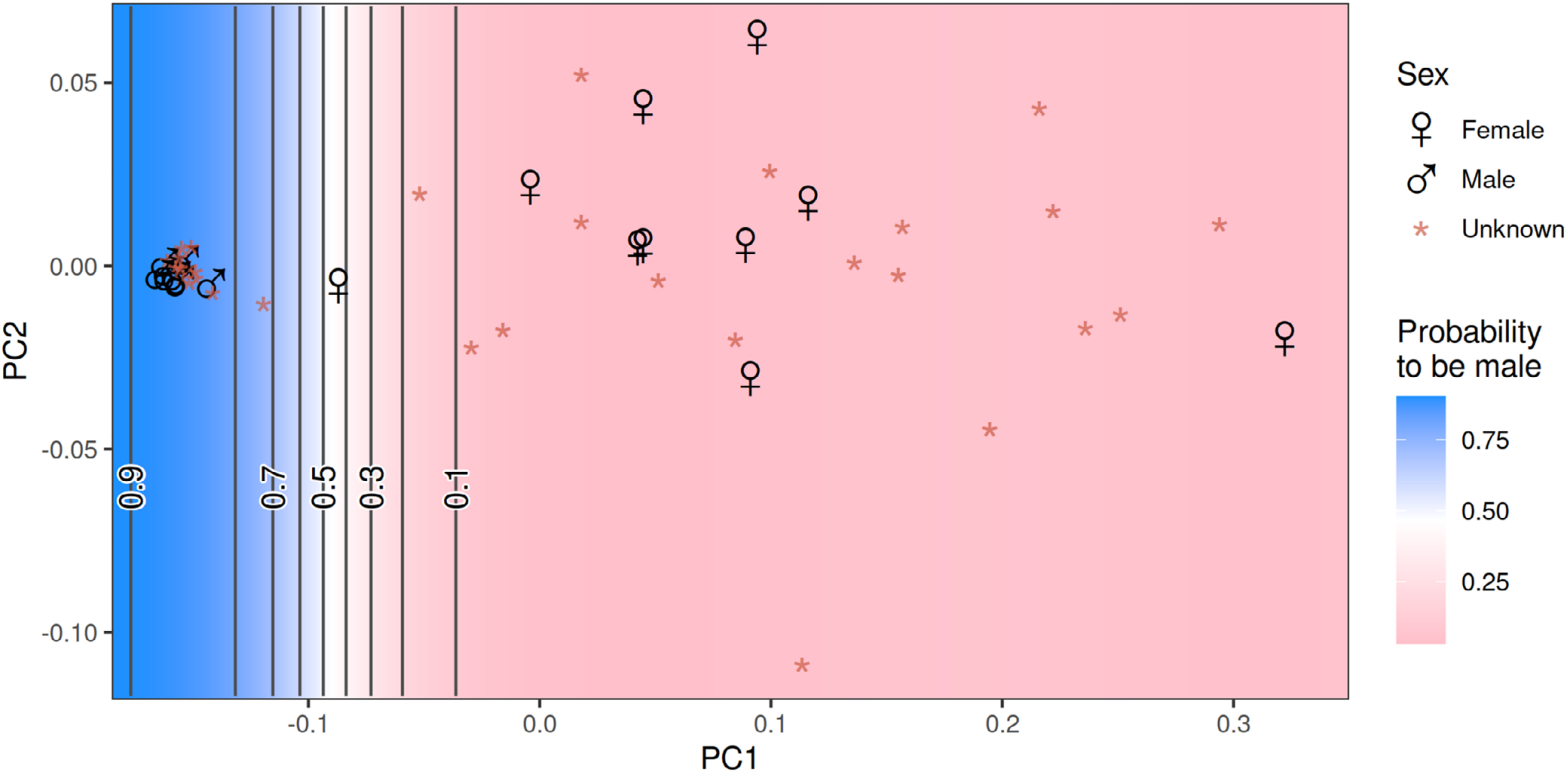
Graph depicting the results of a PCA together with the probabilities of the analyzed individuals to be male indicated by a color gradient. The PCA and the determination of the probabilities with SVM analysis were performed using abundance levels of the Vg isoforms used here as sex markers (Vg isoforms 4, 7, 9; Text S2 with Figures S4 and S5 in the Appendix) in the proteomes of *E. verrucosus* from samplings in September, November, December, and January. The animals with the sex known from their positions in an amplexus (September, November samplings) are indicated with ♀ (female) and ♂ (male) symbols.

The proteomes of *E. verrucosus* individuals with their sex known from their positions in amplexuses were examined for the Vg isoforms showing sex‐specific abundance levels. Ten Vg isoforms were identified in the *E. verrucosus* proteomes (Figure A4, Appendix, and Table S2). Of those, three Vg isoforms (4, 7, 9) showed clear and consistent differences in abundances in female and male proteomes (Figures A4 and A5A in the Appendix). Those Vg isoforms enabled the assignment of sex to nonmating *E. verrucosus* individuals from all sampling months, except for those sampled in June (Figure 1 and Figure A5A,B, Appendix).

### 3.2 Identified Trait‐Specific Proteome Patterns

**FIGURE 2:**
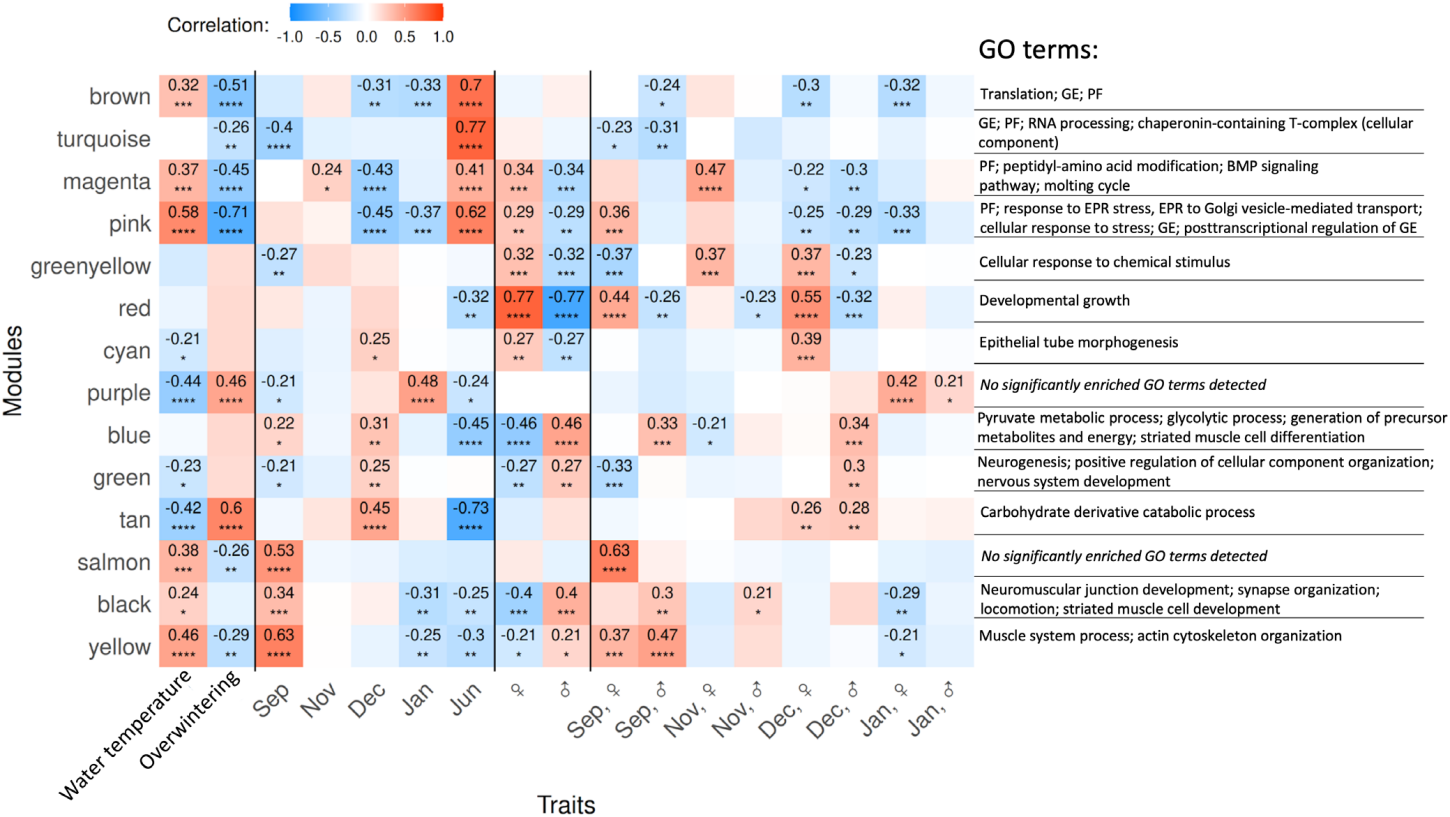
Correlations of WGCNA modules and relevant traits with corresponding significantly enriched gene ontology (GO) terms. The coloring of boxes represents Pearson correlation coefficients (see legend). GO terms relate to the “Biological process” group, unless otherwise indicated. For water temperatures refer to data in Table 1; “Overwintering” refers to the proteins with over the months gradually increasing or decreasing abundance levels in the proteomes of *E. verrucosus* sampled from September to January, independent of the examined animals' sex. Significance levels of correlations: *p ≤ 0.1, **p ≤ 0.05, ***p ≤ 0.01, ****p ≤ 0.001. BMP, bone morphogenetic protein; EPR, endoplasmic reticulum; GE, gene expression; PF, protein folding; ♀, female; ♂, male.

Weighted gene correlation network analysis (WGCNA) was applied to the *E. verrucosus* proteome data obtained here to identify environmental conditions‐related and reproduction‐specific proteome patterns. Using WGCNA, the patterns of co‐abundant proteins in field‐sampled *E. verrucosus* were assigned to 14 color‐coded modules (see Table 2 for the GO terms most highly correlated with each module and the numbers of the identified associated proteins). The obtained module eigengenes (the first principal components of modules) were then examined for their correlations with different traits to identify protein candidates involved in processes related to reproduction or to acclimation to winter conditions. Correlations were analyzed for the parameters “sex” (female, male; all sampling months); “sampling month,” including sex‐specific and non‐sex‐specific proteome pattern characteristics (all sampling months); “overwintering” describing the gradual, non‐sex‐specific proteome pattern changes over the fall/winter period (September to January samplings); and the environmental parameter “water temperature” (water temperatures at the field sampling location during samplings; Table 1). The highest module/trait correlations (*R* ≥ 0.7, *p* < 0.001) were found for the brown and turquoise modules/June and the red module/sex; high correlations (*R* ≥ 0.6, *p* < 0.001) were also seen for the tan module/overwintering, yellow module/September, and salmon module/female in September (Figure 2).

**TABLE 2:** GO terms enriched in the proteins of different modules.

Module	Top GO terms (*p*‐value < 0.01)	Numbers of proteins
Brown	Translation, gene expression, protein folding	45
Turquoise	Gene expression, protein folding, RNA processing, chaperonin‐containing T‐complex (Cellular component)	223
Magenta	Protein folding, peptidyl‐amino acid modification, BMP signaling pathway, molting cycle	58
Pink	Response to endoplasmic reticulum stress, protein folding, endoplasmic reticulum to Golgi vesicle‐mediated transport, cellular response to stress, gene expression, posttranscriptional regulation of gene expression	62
Greenyellow	Cellular response to chemical stimulus	42
Red	Developmental growth	75
Cyan	Epithelial tube morphogenesis	29
Purple	Significantly enriched GO terms were not detected	48
Salmon		34
Blue	Pyruvate metabolic process, glycolytic process, generation of precursor metabolites and energy, striated muscle cell differentiation	173
Green	Neurogenesis, positive regulation of cellular component organization, nervous system development	79
Tan	Carbohydrate derivative catabolic process	40
Black	Neuromuscular junction development, synapse organization, locomotion, striated muscle cell development	68
Yellow	Muscle system process, actin cytoskeleton organization	93

*Note:* The listed GO terms relate to the “Biological process” group, unless otherwise indicated. The column at the right lists the numbers of the identified proteins associated to the different modules.

Gene ontology (GO) terms enriched in the proteins of the different modules comprise processes associated with gene expression and protein turnover, development, and stress response (Figure 2).

### 3.3 Protein Abundance Changes Correlating With the “Overwintering” Trait

**FIGURE 3:**
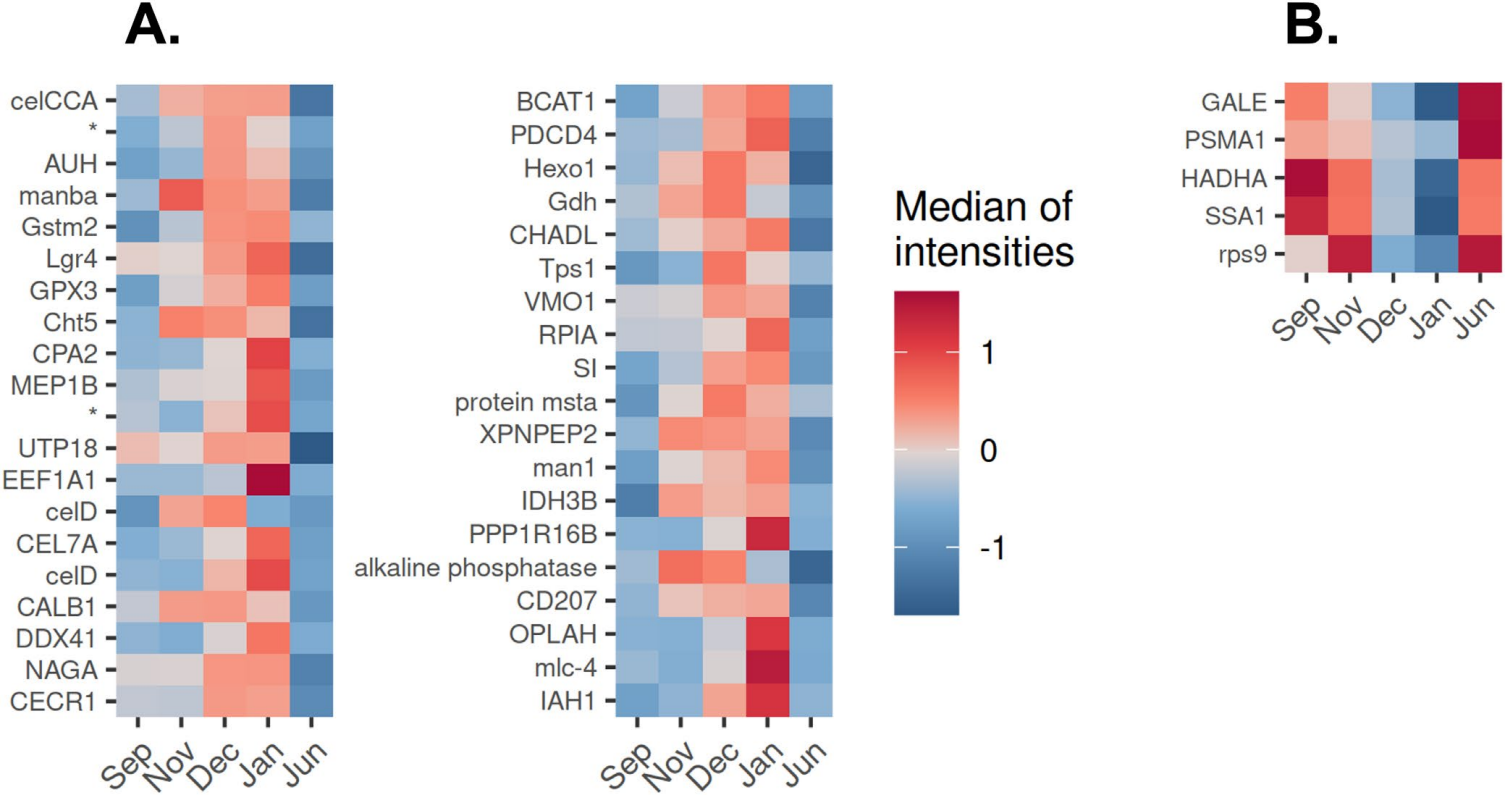
Heatmaps showing median protein abundances (corresponding to the relative intensities) correlating with the “overwintering” trait in *E. verrucosus* (both sexes) across all sampling months. Protein abundances were continuously increasing (A) or decreasing (B) over the fall/winter months (September to January samplings). Protein annotations: See Table S1 (Supplementary Information file). Abundance data for each protein are presented separately for each sex in Figure A6 in the Appendix. For the numbers of examined individuals see Table 1. *—oplophorus‐luciferine 2‐monooxygenase noncatalytic subunit.

**FIGURE A6:**
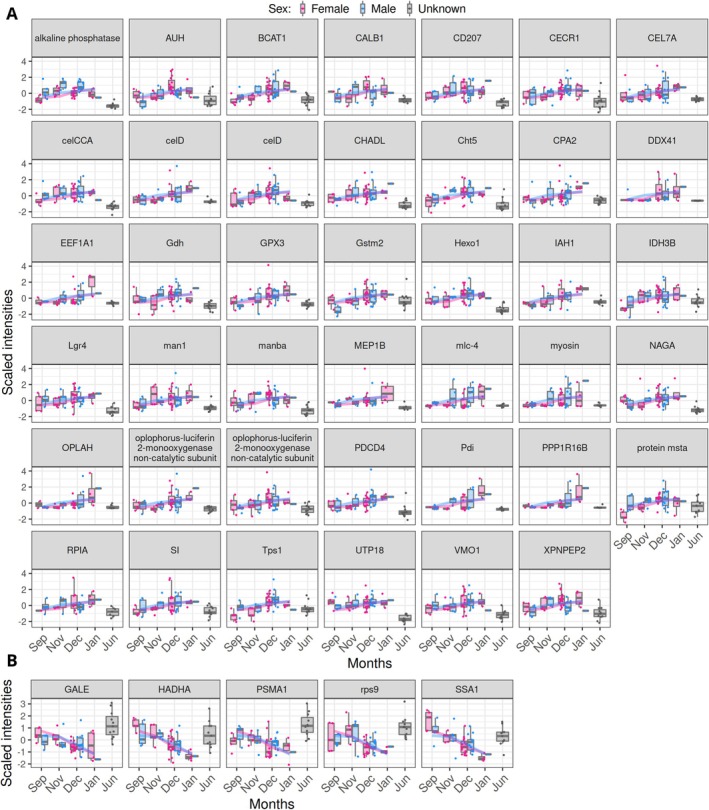
(Supplementary to Figure 3) Proteins showing abundance level changes in the fall and winter sampling months that occurred likewise in both sexes of *E. verrucosus* . Protein abundance levels were continuously increasing (A) or decreasing (B) in *E. verrucosus* sampled from September to January. Protein abundances are shown separately for female (pink) and male (blue) *E. verrucosus* . For clearer representation data for consecutive months are connected with lines in the respective sex‐specific colors.

In the proteomes of *E. verrucosus* of both sexes, abundances of certain proteins of the purple and tan modules exhibited either continuous increases or decreases along the consecutive fall/winter samplings (September to January) with abundance maxima and minima, respectively, occurring in January (Figure 3 and Figure A6, Appendix). Proteins that showed increasing abundances and were annotated with gene significance (GS) levels ≥ 0.45 include DEAD‐Box helicase DDX41, disulfide‐isomerase Pdi, beta‐mannosidase (manba), elongation factor 1‐alpha 1 (EEF1A1), endoglucanases CEL7A, CelCCA, and CelD, the two membrane metallopeptidases Xaa‐Pro aminopeptidase 2 (XPNPEP2) and meprin A subunit beta (MEP1B), carboxypeptidase (CPA2), alkaline phosphatase, branched‐chain‐amino‐acid aminotransferase (BCAT1), methylglutaconyl‐CoA hydratase (AUH), alpha‐N‐acetylgalactosaminidase (NAGA), isoamyl acetate‐hydrolyzing esterase (IAH1), alpha, alpha‐trehalose‐phosphate synthase (Tps1), programmed cell death protein 4 (PDCD4), mitochondrial isocitrate dehydrogenase (NAD) subunit beta (IDH3B), and adenosine deaminase (CECR1) (Figure 3A and Figure A6A, Appendix). Proteins that displayed decreasing abundances over this period include an alpha subunit of mitochondrial trifunctional enzyme (HADHA), a proteasome subunit alpha type‐1 (PSMA1), heat shock protein SSA1, 40S ribosomal protein S9 (Rps9), and UDP‐glucose 4‐epimerase (GALE) (Figure 3B and Figure A6B, Appendix).

Abundance profiles of these proteins positively correlated with the sample trait “overwintering.” Pearson correlation coefficients (*R*) for proteins with increasing abundances were ≥ 0.43 and with decreasing abundances ≤ −0.55. To prevent the inclusion of proteins with the abundance differences solely driven by the sex of the examined individuals, only proteins with no differences in abundance levels between the sexes of the individuals sampled in September and November were considered (*p* value > 0.05; Fisher's combined probability test for two *t*‐test *p* values measuring differences in protein abundances between sexes for September and November).

### 3.4 Female‐Specific Proteome Characteristics

#### 3.4.1 Reproduction Period (September to January Samplings)

**FIGURE 4:**
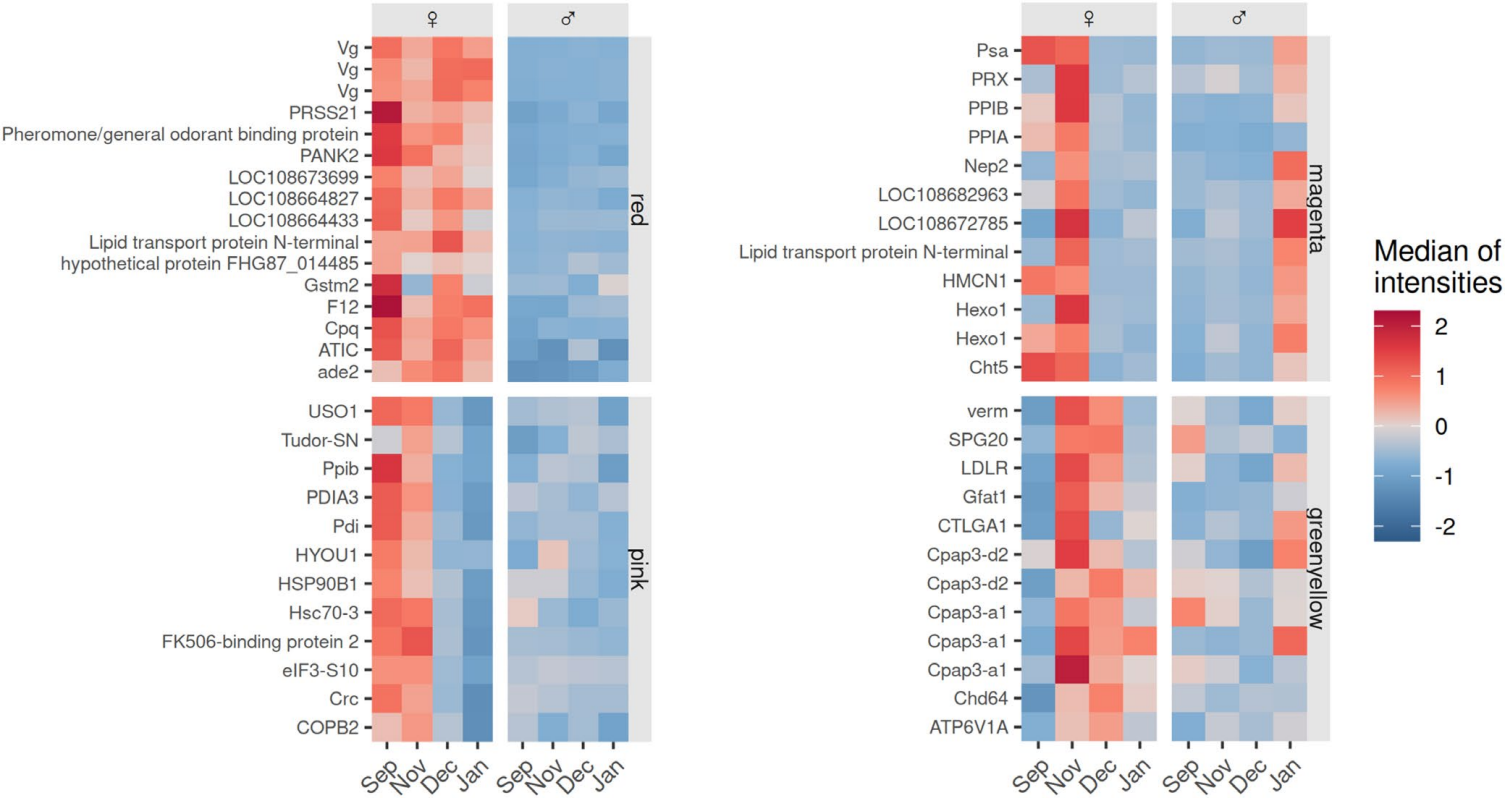
Proteins upregulated in female *E. verrucosus* that were in amplexus when sampled (September and November samplings). Depicted are the 12 most highly abundant proteins of each of the four modules for both *E. verrucosus* sexes for all sampling months. Refer to Table 2 for GO terms associated with each module, to Table S1 (Supplementary Information file) for protein annotations, and for more details on sex‐specific proteome patterns to texts S4–S6 with Figures A8, A9, A10, A11, A12, A13 in the Appendix.

The *red module* includes proteins that can be characterized as female‐specific (Figures 2 and 4) and that were shown to be involved in developmental growth (GO:0048589; Figure 2). These proteins are upregulated in females during all observed months (September, November, December, and January) except for June. Presumably, however, female *E. verrucosus* were among the samples from June, which could not be identified with the molecular sex markers (see above and Text S2 in the Appendix).

**FIGURE A9:**
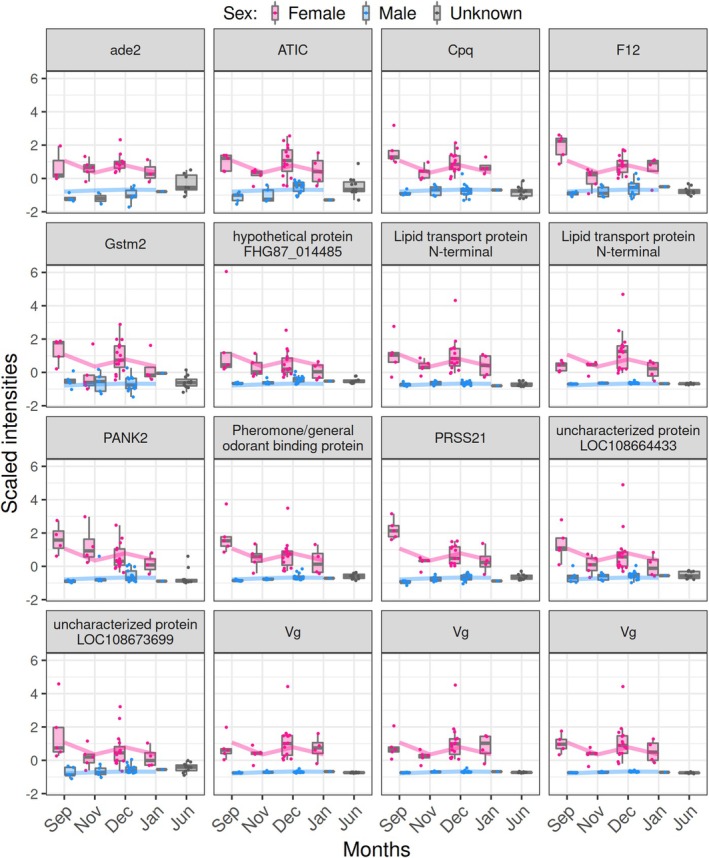
(Supplementary to Figure 4) Abundances of proteins from the female‐specific red module (degree of association to this module > 0.74). Protein abundances are shown separately for the proteomes of female (pink) and male (blue) *E. verrucosus* from the different sampling months. For clearer representation data for consecutive months are connected with lines in the respective sex‐specific colors. The proteins designated as “uncharacterized protein LOC108673699” and “hypothetical protein FHG87_014485” show structural similarities with OBP proteins (for details see section “*(i) Egg protein components”* in Text S5).

Proteins with increased abundances and a degree of association > 0.74 to the red module included phosphoribosylformylglycinamidine synthase ade2, bifunctional purine biosynthesis protein ATIC, carboxypeptidase Q (Cpq), clotting protein F12, glutathione S‐transferase mu 2 (Gstm2), two lipid transport proteins, pantothenate kinase 2 (PANK2), pheromone/general odorant binding protein (OBP), serine protease 21 (Prss21), and Vg isoforms (Figure 4 and Figure A9 in the Appendix). For two proteins of this module, the highest matches of a BLAST (NCBI Basic Local Alignment Search Tool) run were “uncharacterized protein LOC108673699” and “hypothetical protein FHG87_014485” (Figure 4 and Figure A9 in the Appendix). However, the two proteins could also be annotated with high confidence as pheromone/general odorant binding proteins (OBPs), as they showed high similarities with respective orthologs of the amphipod *Trinorchestia longiramus* (*E*‐values of 2·10^−34^ and 1·10^−40^ for “uncharacterized protein LOC108673699” and “hypothetical protein FHG87_014485”, respectively).

**FIGURE A10:**
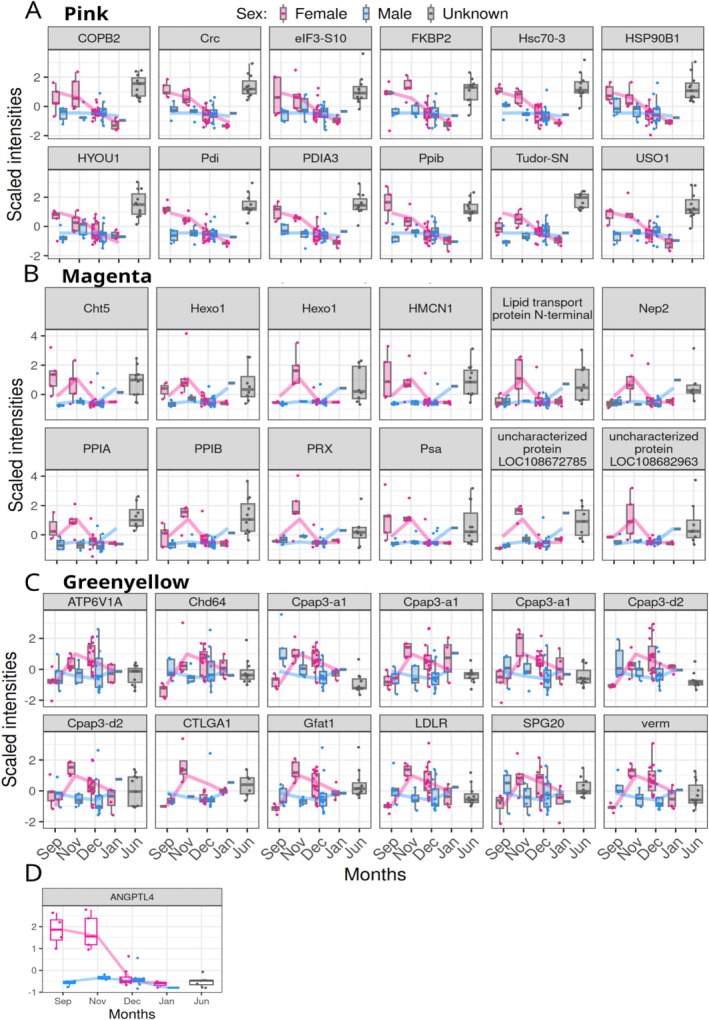
(Supplementary to Figure 4) Abundances of proteins upregulated in female *E. verrucous* when in amplexus (September, November samplings). Protein abundances are shown separately for the proteomes of female (pink) and male (blue) *E. verrucosus* from the different sampling months. For clearer representation data for consecutive months are connected with lines in the respective sex‐specific colors. The proteins are associated with the pink (A), magenta (B), and greenyellow modules (C); for each module, the abundances of the twelve most highly abundant proteins are shown. (D) Protein abundance levels of ANGPTL4, which were exceptionally high and are therefore depicted here. Refer to Table 2 for details on the different modules.

**FIGURE A11:**
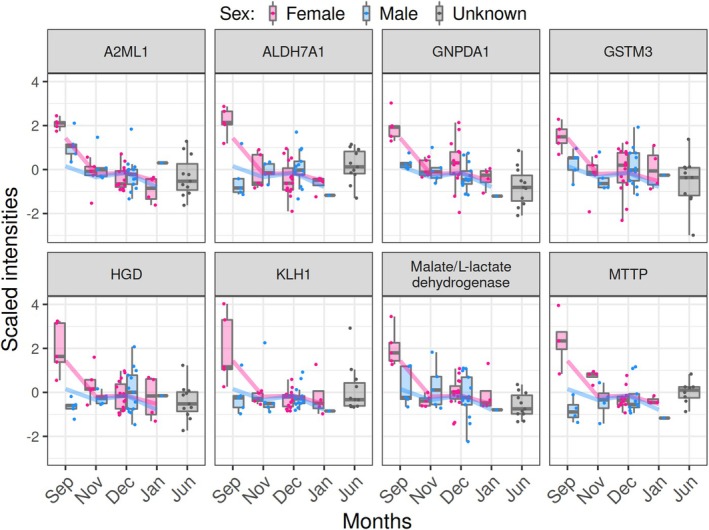
(Supplementray to the section “Female‐specific proteome characteristics”) Levels of proteins with abundance peaks in the proteomes of female *E. verrucosus* sampled in September and with the highest positive correlations to the salmon module [MM (model membership) > 0.5; *p* < 0.05; *t*‐test for females vs. males in September]. Differences in the abundances of those proteins between the sexes were significant (*p* < 0.05; *t*‐test for females vs. males in September). Protein abundances in samples from the different sampling months are shown separately for female (pink) and male (blue) *E. verrucosus* .

Other highly abundant proteins in female *E. verrucosus* during this period included angiopoietin‐related protein ANGPTL4 (Figure A10D, Appendix), the large subunit of microsomal triglyceride transfer protein MTTP (Figure A11, Appendix), chitinase Cht5, aminopeptidase Psa, and hemicentin HMCN1 (Figure A10B, Appendix). More information on the highly abundant proteins is provided in Text S4 and Figure S8 in the Appendix.

#### 3.4.2 Females in Amplexus (September, November Samplings)

The proteome patterns of female *E. verrucosus* that were in amplexus when sampled could be assigned to the pink, magenta, and greenyellow and salmon modules (Figure 4; Figures A10 and A11 in the Appendix).

Proteins of the *pink module* are associated with GO terms “response to endoplasmic reticulum stress” (GO:0034976), “protein folding” (GO:0006457), and “cellular response to stress” (GO:0033554) (Figure 2; see also Figure 4 and Figure A10A in the Appendix). This module includes proteins involved in maintenance of proteostasis, such as chaperones Hsc70‐3 and Hsp90B1, calreticulin (Crc), protein disulfide‐isomerases Pdi, PDIA3, Ppib, peptidyl‐prolyl cis‐trans isomerase FKBP4; and a protein involved in response to hypoxia, the hypoxia upregulated protein 1 (HYOU1). Two proteins of this module, the coatomer subunit beta (COPB2) and general vesicular transport factor p115 (USO1), are involved in endoplasmic reticulum‐Golgi vesicle transport.

Many proteins of the *magenta* and *greenyellow modules* were highly abundant, especially in samples from November the magenta module includes proteins related to peptidyl‐amino acid modification (GO:0018193; Figure 2), such as neprilysin‐2 (Nep2) and aminopeptidase Psa, that are involved in peptide catabolic processes, and two peptidyl‐prolyl cis‐trans isomerases (PPIA and PPIB). Furthermore, several highly abundant proteins of this module are involved in chitin degradation (GO:0042303, molting cycle), such as two isoforms of chitooligosaccharidolytic beta‐N‐acetylglucosaminidase Hexo1 and chitinase 5 (Cht5) (Figure 4 and Figure A10B in the Appendix).

Proteins of the *greenyellow module* are related to functions in the molting cycle, such as proteins from the Cpap3 family involved in cuticle formation (Abehsera et al. 2018) and vermiform, involved in chitin organization in the cuticle (Wang et al. 2006). Proteins with high abundances associated with other functions within this module are glutamine‐fructose‐6‐phosphate transaminase 1 (Gfat1), spartin protein Spg20, and low‐density lipoprotein receptor (LDLR) (Figure 4 and Figure A10C in the Appendix).

The *salmon module* includes proteins that show abundance peaks in the proteomes of the animals sampled in September. Among the most highly abundant proteins in this module were hemocyanin KLH1, alpha‐aminoadipic semialdehyde dehydrogenase ALDH7A1, glucosamine‐6‐phosphate isomerase 1 GNPDA1, and the large subunit of the microsomal triglyceride transfer protein (MTTP) (Figure A11, Appendix).

Abundance levels of angiopoietin‐related protein 4 (ANGPTL4), with no significant association to any of the above modules, showed abundance levels that were among the highest in female *E. verrucosus* in amplexus (see Text S5(iii) and Figure A10D in the Appendix).

### 3.5 Male‐Specific Proteome Characteristics

Proteins from the black and blue modules showed increased abundances in the male *E. verrucosus* sampled in amplexus (September and November; Figure A13 in the Appendix). Highest abundances were seen for proteins associated with GO terms related to energy production and anabolic processes in muscle cells: pyruvate metabolic process (GO:0006090), generation of precursor metabolites and energy (GO:0006091), glycolytic process (GO:0006096), striated muscle cell differentiation (GO:0051146), striated muscle cell development (GO:0055002), and neuromuscular junction development (GO:0007528; Figure 2). Furthermore, some of the more highly abundant proteins are known to function as structural components of the sarcomere, such as four isoforms of twitchin protein, titin (sls), two isoforms of myosin heavy chain, muscle LIM protein Mlp84B, paramyosin Prm, muscle‐specific protein 300 kDa (Msp300), muscle M‐line assembly protein unc‐89, and myozenin (Figure A13, Appendix). Further proteins showing upregulation were eIF‐2‐alpha kinase activator GCN1, activated in response to amino acid starvation and triggering amino acid synthesis (Hinnebusch 1994); a mitochondrial form of aspartate aminotransferase (GOT2), involved in amino acid metabolism; and the catalytic subunit of calcineurin (PPP3CC) (Figure A13, Appendix). Glycolysis‐related proteins were found to be upregulated particularly in September, such as fructose‐6‐phosphate aldolase (ALDOC), phosphoglycerate mutase (PGAM2), phosphoglycerate kinase (Pgk), ATP‐dependent 6‐phosphofructokinase (Pfk), triosephosphate isomerase TPI1, and pyruvate kinase PyK (Figure A13, Appendix).

The proteomes of males in amplexus also showed increased abundances of proteins involved in cytoskeleton formation, such as ankyrin‐3 (ANK3), F‐actin‐capping protein subunit beta (CAPZB), nesprin‐1 (SYNE1), and filamin B (FLNB) (Text S6 and Figure S13 in the Appendix).

## 4 Discussion

### 4.1 Acclimation to Season‐Related Environmental Conditions

Baikal's littoral gammarids face pronounced daily and seasonal fluctuations within environmental parameters, including temperature, food abundance, and daylight length (Figure 6; Timoshkin 2018). In the summer, sudden changes in water currents following weather changes can cause considerable short‐term fluctuations of water temperatures in the littoral (Tsimitri et al. 2015, see also Figure A1 in the Appendix). Distinct molecular, biochemical, and physiological acclimation processes, as well as behavioral responses, enable Baikal's littoral amphipods to tolerate water temperature changes (Drozdova et al. 2019; Jakob et al. 2016, 2021; Lipaeva et al. 2023; Vereshchagina et al. 2021).

Differential abundances of several proteins in *E. verrucosus* from the fall and the beginning of winter samplings indicate acclimation responses of both sexes likewise (i) to decreasing water temperatures and (ii) to changes in diet:

DEAD‐Box helicase DDX41 and disulfide‐isomerase Pdi (Text S3, Figure A6A in the Appendix) can be considered as candidate proteins involved in the acclimation to decreasing temperature. Previously, a group of DEAD‐Box helicases was also found to be upregulated in the amphipod *G. lacustris* upon exposure to low water temperature in the laboratory (Lipaeva et al. 2021). The function of DEAD‐Box helicases, found to be enriched in prokaryotes and eukaryotes upon cold stress, was associated with the untangling of misfolded RNA (Gracey et al. 2004; Guan et al. 2013; Hunger et al. 2006; Yang et al. 2014). Disulfide‐isomerase Pdi may function as a chaperone stabilizing proteins upon disturbances of hydrophobic interactions within proteins by various stressors, including cold stress (Trivedi et al. 2016); the upregulation of disulfide‐isomerase in an Arctic springtail upon cold stress (Thorne et al. 2011) may be seen in this context.

Abundance changes of various metabolic enzymes in *E. verrucosus* during the fall–winter transition may be due to acclimation responses to an altering diet but, especially in the case of enzymes of lipid catabolic processes, may also be a response to declining temperatures to support homeoviscous adaptation, that is, maintenance of adequate membrane fluidity. Respective candidates were proteins involved in cellulose degradation (glucanases Cel7a, CelCCA, and CelD); in lipid catabolic processes (alpha‐N‐acetylgalactosaminidase NAGA and isoamyl acetate‐hydrolyzing esterase IAH1); in the protein catabolic process (carboxypeptidase CPA2); and in purine catabolism (adenosine deaminase CECR1) (Figure A6A, Appendix). These proteins were found to be expressed in *E. verrucosus* hepatopancreas (own unpublished data), supporting their function of enzymatic processing of ingested food. Abundance changes of branched‐chain amino acid aminotransferase BCAT1 (Figure A6A, Appendix), which initiates the catabolism of branched‐chain amino acids (leucine, isoleucine, valine) (Toyokawa et al. 2021), may also be food‐related. With respect to homeoviscous adaptation, enzymes of lipid catabolic processes that break down glycolipids could be involved, which play a role in maintaining the stability of cellular membranes (Bauersachs et al. 2015).

### 4.2 Proteome Patterns Triggered by Reproduction‐Related Processes

**FIGURE 5:**
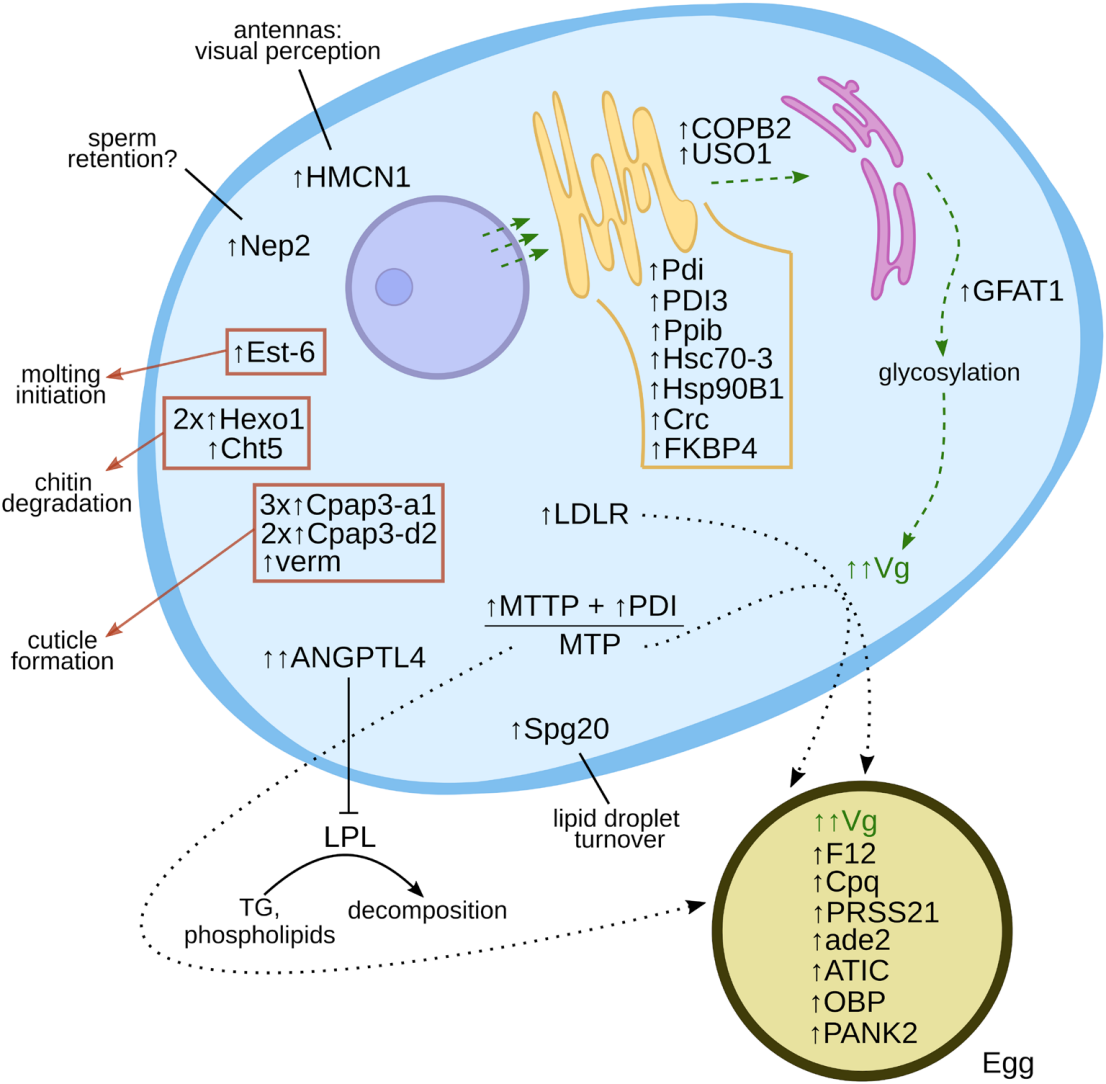
Upregulated proteins and the respective reproduction‐related processes in female *E. verrucosus* in winter. ade2, phosphoribosylformylglycinamidine synthase; ANGPTL4, angiopoietin‐related protein; ATIC, bifunctional purine biosynthesis protein; Cht5, chitinase; COPB2, coatomer subunit beta; Cpap3, cuticular protein; Cpq, carboxypeptidase Q; Crc, calreticulin; Est‐6, carboxylesterase; F12, clotting protein; FKBP4, peptidyl‐prolyl cis‐trans isomerase; GFAT1, glutamine‐fructose‐6‐phosphate transaminase 1; Hexo1, chitooligosaccharidolytic beta‐N‐acetylglucosaminidase; HMCN1, hemicentin; Hsc70‐3, heat shock 70 kDa protein 3; Hsp90B, heat shock protein HSP 90‐beta; LDLR, low‐density lipoprotein receptor; LPL, lipoprotein lipase; MTP, heterodimeric protein complex MTP; MTTP, the large subunit of microsomal triglyceride transfer protein; Nep2, neprilysin‐2; OBP, pheromone/general odorant binding protein; PANK2, pantothenate kinase; PDI, disulfide‐isomerase; Ppib, peptidyl‐prolyl cis‐trans isomerases; PRSS21, serine protease; Spg20, spartin protein; TG, triglycerides; USO1, general vesicular transport factor p115; verm, vermiform; Vg, vitellogenin.

**FIGURE A12:**
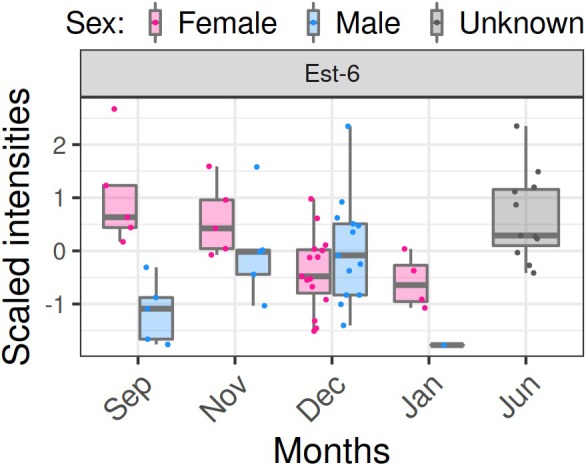
(Supplementary to Figure 5) Abundances of the juvenile hormone esterase‐like carboxylesterase Est‐6 in the proteomes of *E. verrucosus* from the different sampling months. Data are presented separately for female and male individuals, except for June when sexes could not be distinguished. Pink and blue fat vertical lines in the box plots represent the means of all obtained abundance values for a given month.

**FIGURE A13:**
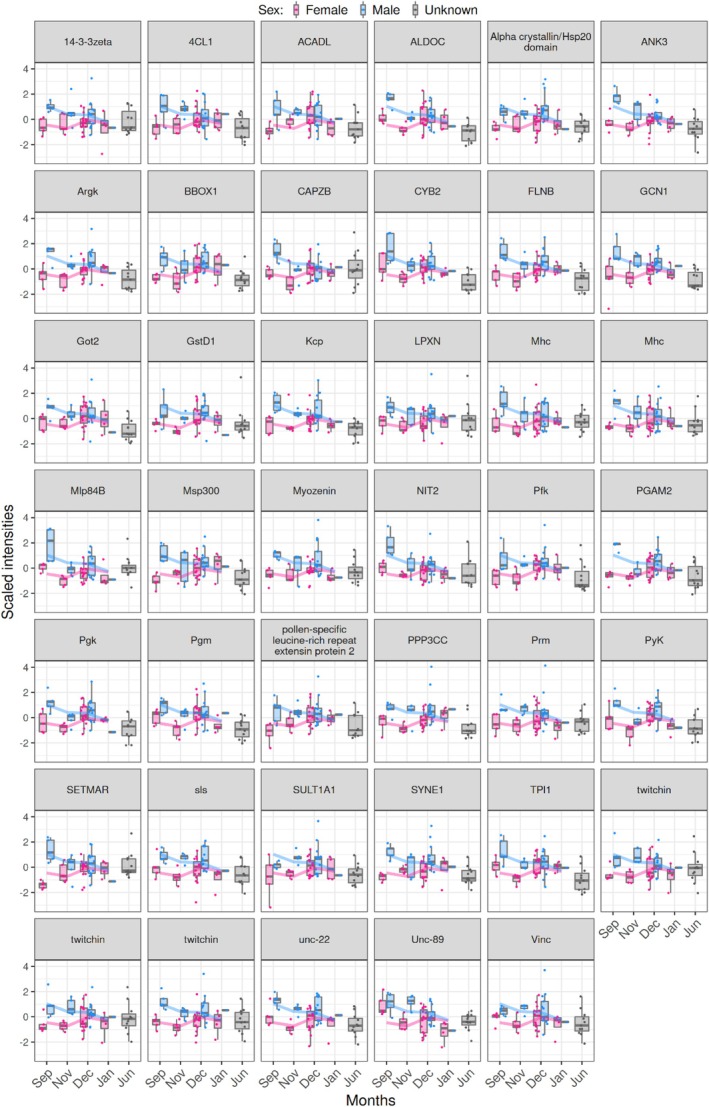
(Supplementray to the section “Male‐specific proteome characteristics”) Abundances of proteins upregulated in male *E. verrucous* that were sampled when in amplexus (September and November samplings). Protein levels are shown separately for both sexes of *E. verrucosus* in the different sampling months. Proteins with *p*‐values < 0.009 (Fisher's combined probability test for the two *t*‐test *p*‐values for the differences in protein abundances between the sexes in September and November) are depicted. For clearer representation, data for consecutive months are connected with lines in the respective sex‐specific colors.

Our analyses of whole‐organism proteomes showed clear sex‐specific patterns resembling those of the summer‐reproducing Baikal amphipod *E. cyaneus* (Bedulina et al. 2021) and also similar to those found for the reproductive organs of the amphipod *Gammarus fossarum* (Trapp et al. 2016). The sex‐specificity of whole‐organism proteomes mirrors the predominance of reproduction‐related processes in amphipods in the respective times of the year. Sexual dimorphism of proteomes is predominantly due to the upregulation of reproduction‐related female‐specific proteins during the reproduction period (Figure 5; Figure A9, Appendix). In the time between reproductive cycles (June for *E. verrucosus* ) female amphipods are in a state of ovarian diapause (Sutcliffe 1992), and the sex‐specificity of proteomes of *E. verrucosus* sampled in June was not obvious (data for June in Figures A5B and A9, A10, A11, A12, A13 in the Appendix). Female proteomes show two categories of reproduction‐related characteristics, involving proteins of (i) oocyte‐related metabolic processes and acting as oocyte/egg constituents and of (ii) processes in females related to: increased oxygen demand in the amplexus state (September); molting prior to fertilization (Borowsky 1984); sperm retention (neprilysin‐2 [Sitnik et al. 2014]); and impairment of vision (hemicentin; [Liberti et al. 2019]) (Figure 5; refer to Text S5 in the Appendix for *E. verrucosus* female‐specific protein abundances).

### 4.3 Timing of Reproduction‐Related Processes and Environmental Parameters

**FIGURE 6:**
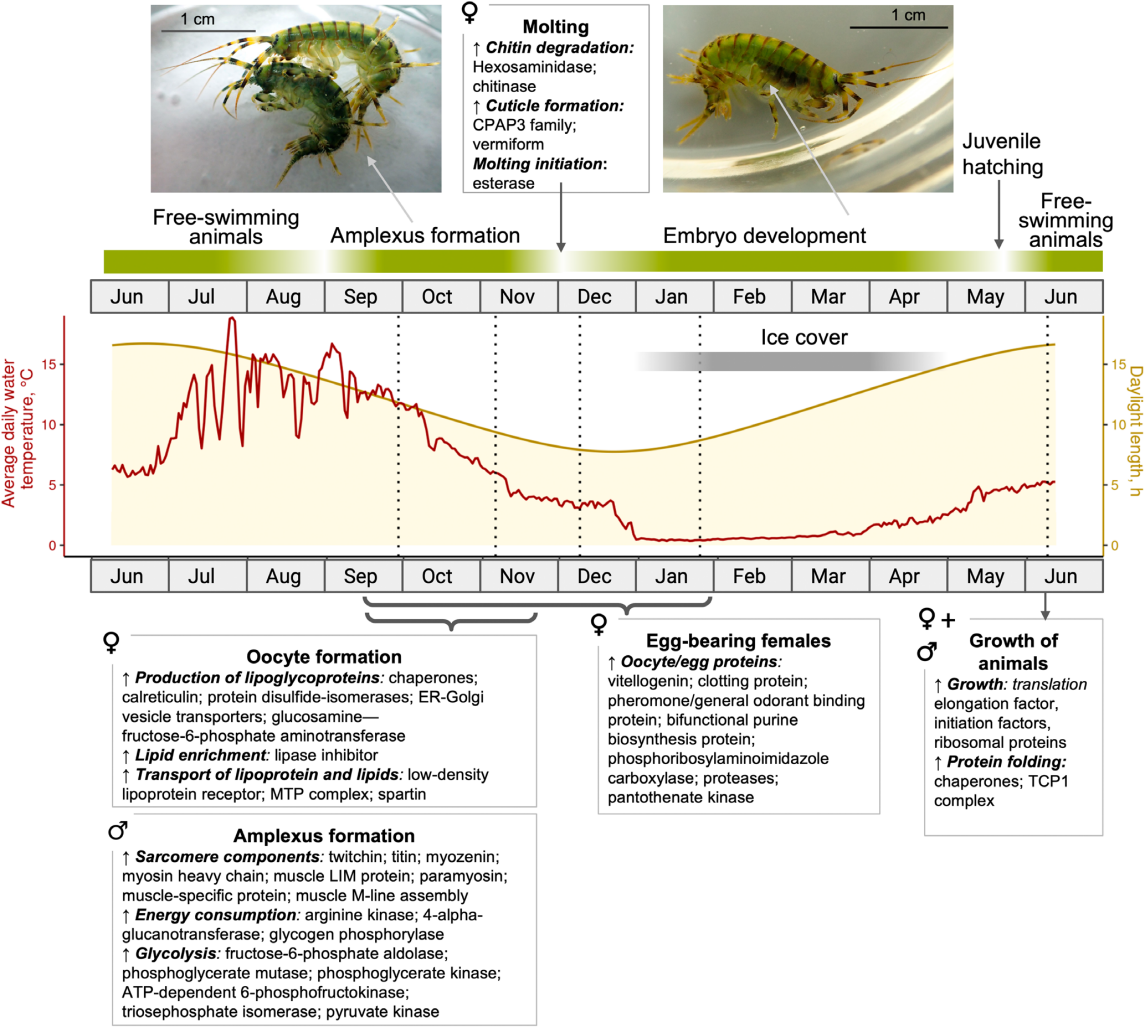
Timeline of events and processes related to reproduction in *E. verrucosus* and of relevant season‐dependent environmental parameters. Those include water temperature, daylight length (courses of both parameters indicated by continuous lines), and the time period of ice cover (gray bar). Parameter data are for the region of the *E. verrucosus* sampling location [Bolshie Koty bay at Lake Baikal; see Materials and Methods and Figure A1 in the Appendix for a description of how temperature data were obtained and for a more detailed presentation of those data]. Times of reproduction‐related processes (mating of parental animals/amplexus formation, embryo development, hatching of juveniles from eggs) are illustrated with green lines above the graph. “Free‐swimming animals”: *E. verrucosus* outside of the reproductive season. Field sampling time points are indicated by dotted lines. Photographs depict an *E. verrucosus* mating pair (amplexus; left) and an *E. verrucosus* female with developing embryos in her brood pouch (white arrow). The depicted time period covers one year, starting in June 2019; values/times of occurrence of the shown environmental parameters can be seen as exemplary for the respective time of the year. ♀, female; ♂, male.

The timing of endogenous physiological processes deduced from the proteomes of field‐sampled *E. verrucosus* was aligned with the times of occurrences of the amphipods' life‐stage specific events and with relevant environmental parameters (Figure 6). Occurrences of life stage‐specific processes and states “oocyte‐formation in females,” “amplexus‐formation,” “oocyte/egg‐bearing females,” and “growth of hatched animals” were deduced from proteome patterns. Designations of the following periods are based on observations (Bazikalova 1941): “free‐swimming animals”— *E. verrucosus* juveniles after hatching from the eggs and adults that were not in amplexus; “amplexus formation”— *E. verrucosus* adults forming mating pairs; “embryo development”—life stages inside the egg from fertilization to hatching; “juvenile hatching”—hatching from the eggs. Notably, processes that require the allocation of external energy and resources, such as oocyte formation and mate finding/amplexus assembly, take place before the onset of winter conditions. In contrast, embryo development, which proceeds over several months during winter/early spring, does not depend on the availability of external food but is supplied by the egg yolk reserves. Juvenile *E. verrucosus* hatch from the eggs during the transition from winter to summer conditions. For growth, the juveniles thus can benefit from higher temperatures and increased availability of external resources. Data on the abundance of microphytobenthos (Pomazkina et al. 2008; Figure A2, Appendix) indicate the availability of external food resources for *E. verrucosus* , which tends to be higher in the summer than in the winter months. The study on microphytobenthos abundance in the littoral was performed at another time than our study, but the data by Pomazkina et al. (2008) may nevertheless be representative. Daylight length as a potential trigger for time‐of‐the‐year‐related processes in the amphipods (Sutcliffe 1992) was included in Figure 6.

## 5 Conclusions

Considering the harsh winter conditions in Lake Baikal, it appears remarkable that amphipods reproducing during winter form a dominant, species‐rich complex in the lake's littoral. In addition to the amphipods' ability to adjust to low temperatures on the molecular and physiological levels, their specific adaptations also include the pace and the timing of the reproduction‐related processes. The integrative proteomics approach presented here enabled the identification of proteomic hallmarks of reproduction‐related processes and of acclimation to season‐related changes in environmental conditions in the Baikal littoral. As illustrated here with one representative species, Baikal's winter‐reproducing amphipods are characterized by an extended reproduction cycle from fall until the following summer. In contrast, Baikal's littoral summer‐reproducing amphipods have two offspring cohorts per season. Yet, reproduction during the winter appears to be advantageous, considering the dominance of the respective amphipod species complex in Baikal's littoral. Thus, oocyte/egg‐bearing females may be less prone to predation and thus less vulnerable during winter. Baikal's unique environmental conditions, characterized by a constantly liquid and oxygen‐rich water column, are a precondition enabling the amphipods to remain metabolically active and to reproduce during winter. A key benefit of the here‐revealed timing strategy of reproduction‐related processes is the avoidance of concomitance of the occurrence of adverse environmental conditions (low temperature, low food abundance) and resource‐demanding metabolic processes.

## Author Contributions

**Polina Lipaeva:** data curation (lead), investigation (lead), methodology (lead), visualization (lead), writing – original draft (lead). **Polina Drozdova:** data curation (supporting), investigation (supporting), methodology (supporting), supervision (supporting), writing – original draft (supporting), writing – review and editing (supporting). **Kseniya Vereshchagina:** investigation (lead), supervision (equal), writing – original draft (supporting), writing – review and editing (supporting). **Lena Jakob:** data curation (lead), investigation (lead), writing – review and editing (supporting). **Kristin Schubert:** methodology (lead), supervision (lead), writing – review and editing (supporting). **Daria Bedulina:** conceptualization (equal), formal analysis (supporting), funding acquisition (equal), investigation (supporting), project administration (equal), supervision (supporting), writing – original draft (supporting), writing – review and editing (supporting). **Till Luckenbach:** conceptualization (equal), funding acquisition (equal), project administration (equal), supervision (equal), writing – original draft (equal), writing – review and editing (lead).

## Conflicts of Interest

The authors declare no conflicts of interest.

## Supporting information


Table S1.

Table S2.

## Figures and Tables

**FIGURE A7:**
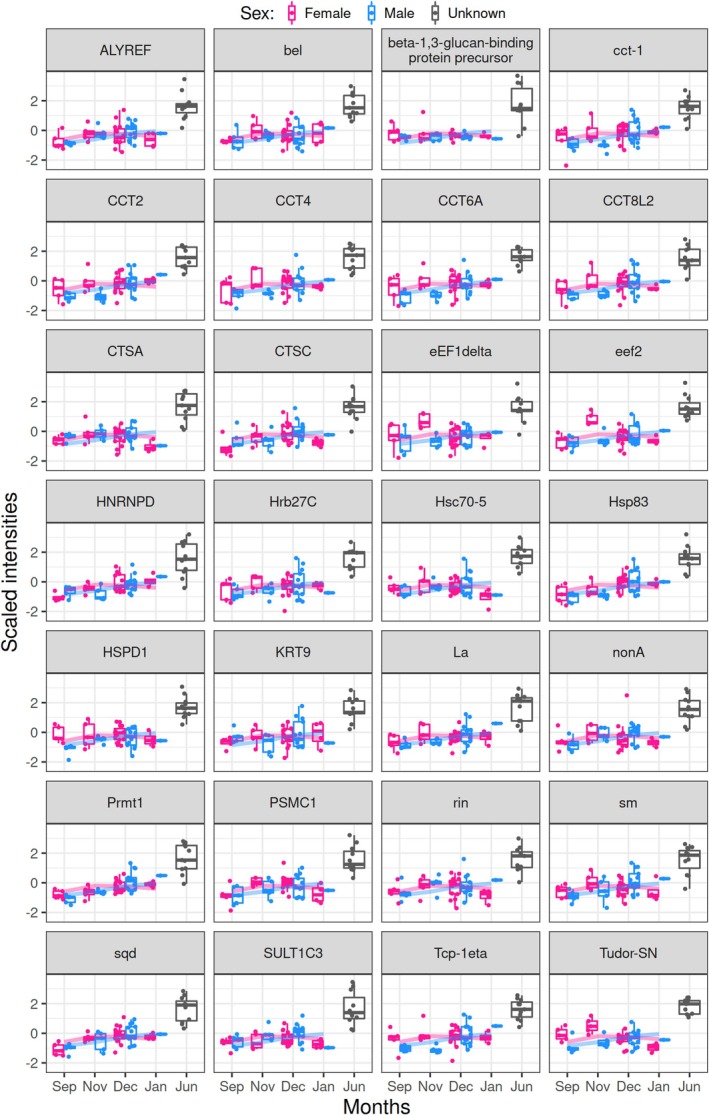
(Supplementary to Figure 2) Abundances of proteins showing a trend to be upregulated in summer. In the proteomes of *E. verrucosus* from the consecutive samplings from September on, the abundances of those proteins showed increasing trends, with peaks in the samples from June (scaled intensity ≥ 0.7). The pink/blue lines in the boxplots represent mean abundance levels of the respective proteins for the given month. For clearer representation, data for *E. verrucosus* field‐sampled in consecutive months are connected with lines in the respective sex‐specific colors.

**FIGURE A8:**
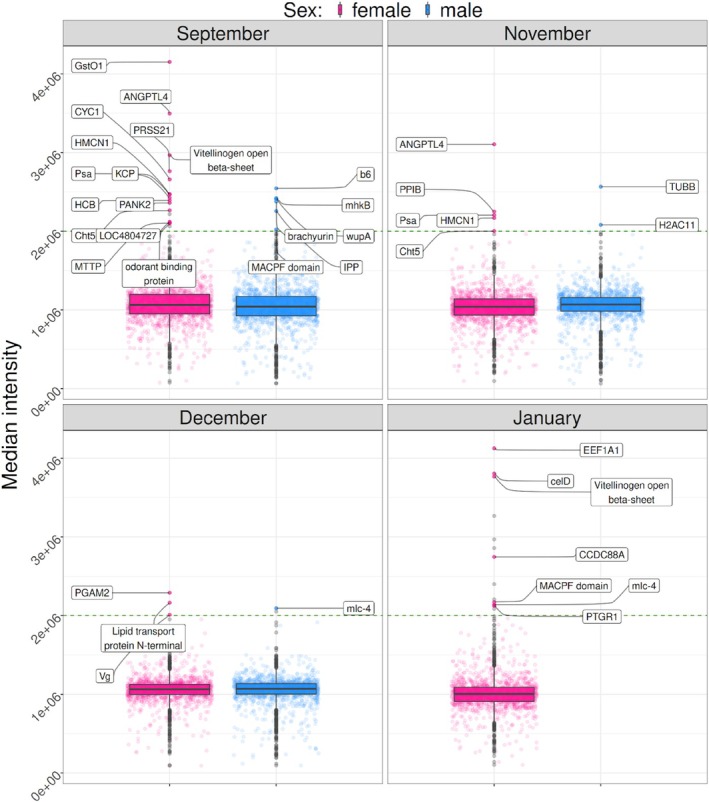
(Supplementray to the section “Female‐specific proteome characteristics”) Protein abundance levels in *E. verrucosus* samples from different months, with highly abundant proteins (≥ 2 × 10^6^ median intensity; marked by dots above the dashed green line) highlighted. Highly abundant proteins with a known function were labeled; dots were not labeled if the protein function was unknown. Each dot represents the median signal intensity for a protein obtained for all individuals of a specific sex sampled in a certain month. Data points are shown only for proteins, for which abundance data were retrieved in at least three replicates. (A) Data for female and male *E. verrucosus* from September, November, December, and January samplings. For January, data were obtained from only one male individual, and a boxplot was not created. (B) Data for *E. verrucosus* individuals sampled in June. The sex of the animals could not be determined and data for all animals from the June sampling are shown in one plot.

## Data Availability

The here obtained MS proteomics data have been deposited in the ProteomeXchange Consortium via the PRIDE (Perez‐Riverol et al. 2022) partner repository with the dataset identifier PXD040677 and 10.6019/PXD040677.

## Text S1. Details on Proteomics Data Acquisition, Processing, and Analysis (Supplementary to “Acquisition and Processing of Proteomics Data” in Materials and Methods)

Complete frozen animals were individually ground to powder with a pre‐chilled mortar and pestle in liquid nitrogen with 50–100 μL extraction buffer (100 mM ammonium bicarbonate with protease inhibitors) and 2—6 μL 10% PMSF. The tissue powder with the buffer was incubated on ice for at least 15 min and then centrifuged at 7000 rpm for 15 min at 4°C. The supernatant was mixed with lysis buffer (200 mM ammonium bicarbonate, 8% SDS, 50 mM TCEP, and protease inhibitors) at a ratio of 1:1 and incubated at 95°C for 3 min. The protein concentration was measured using the Pierce 660 nm Protein Assay Kit with ionic detergent compatibility reagent (Thermo Fisher Scientific).

Tandem mass tag (TMT)‐labeling (TMT‐10‐plex, Thermo Scientific, Waltham, MA) was used to enable multiplexed protein identification and quantitative analysis of proteomes. Sample preparation (reduction by 200 mM TCEP, alkylation by 375 mM IAA, and digestion with 1 μg/μL trypsin) and TMT‐labeling were performed on paramagnetic beads using the SP3 protocol (Hughes et al. 2014; Wang et al. 2021). Per sample, 20 μg of protein were used.

Samples were analyzed on a nano‐UPLC system (Ultimate 3000, Dionex, USA), coupled online via a chip‐based ESI source (Nanomate, Advion, USA) to a mass spectrometer (QExactive, Thermo Scientific, USA). After trapping (Acclaim PepMap 100 C18, 3 μm, nanoViper, 75 μm × 5 cm, Thermo Fisher, Germany), peptides were separated on a reversed‐phase column (Acclaim PepMap 100 C18, 3 μm, nanoViper, 75 μm × 25 cm, Thermo Fisher, Germany), applying a non‐linear gradient of 150 min.

### Peptide Assembly and Data Normalization

The obtained MS raw data were processed using MaxQuant v1.6.17.0 with isobaric matching between runs and peptide‐spectrum match level normalization (Yu et al. 2020). Peptide searching was performed against a customized database with previously obtained transcriptome data from three amphipod species, *E. verrucosus*, related *Eulimnogammarus cyaneus*, and *Gammarus lacustris*. The transcriptome database was built with de novo assembled raw mRNA sequencing data deposited in the NCBI BioProject database (https://www.ncbi.nlm.nih.gov/bioproject/) and have the BioProject accession number PRJNA660769 (https://www.ncbi.nlm.nih.gov/bioproject/?term=660769).

Transcriptome assemblies were performed with rnaSPAdes (Bushmanova et al. 2019), the package from SPAdes v3.14.1 with ‐‐ss fr parameter that indicates the strand specificity of the libraries, and with Trinity (Grabherr et al. 2011), version 2.9.1 with the ‐‐SS_lib_type FR parameter. The RNAspades and Trinity assemblies for each species were combined and filtered with cd‐hit (Li and Godzik 2006), v4.8.1, with the sequence identity threshold parameter set to ‐c 1 to avoid redundant sequences. The final database contained the assembly data for all three species, which were filtered with cd‐hit with the paramTraiteters set as mentioned above. In total, 2127 proteins were identified in *E. verrucosus* proteomes. Data were further processed by excluding proteins (1) that were annotated based only on one peptide; (2) that had less than four non‐zero values in at least one condition in the proteomes of animals directly frozen after field sampling.

For further analysis, corrected reporter intensities were extracted from the proteinGroup text file generated by MaxQuant. The analysis of intensity values was performed with in‐house scripts (https://github.com/PolinaLip/SeasonalExp_Proteome_BaikalAmphipods). To correct the samples for sample loading, sample loading normalization was performed by scaling the total reporter ion intensity for each sample to the average total intensity across all samples (Plubell et al. 2017).

## Text S2. Vitellogenin Isoforms as Molecular Sex Markers

### Background

As candidate for a molecular sex marker we chose vitellogenin (Vg) abundance levels. The Vg isoform levels were analyzed in the proteomes of *E. verrucosus* individuals that were in amplexus when sampled (September, November samplings) to identify the ones clearly indicating the sex of an *E. verrucosus* individual.

### Materials and Methods

*Eulimnogammarus verrucosus* proteomes were obtained and analyzed as described in Materials and Methods (main text) and in Text S1.

### Results

From ten different proteins annotated as Vg and classified as different Vg isoforms (Table S2, provided as SI file), three showed clearly different abundances in the proteomes of female and male *E. verrucosus* (Figures A4 and A5A); abundance levels of those Vg isoforms in proteomes of females were > 2‐fold above those from male *E. verrucosus* from September and November samplings (Figure A5A). Abundances of those Vg isoforms were thus used as molecular sex markers for *E. verrucous* that were not in amplexus when sampled. A Support Vector Machine (SVM) (R package e1071 version 1.7‐9) was trained on the Vg abundance values in order to identify sexes of animals from December and January (Figure A5). The sex was assigned to samples based on the labels predicted by the SVM. A principal component analysis (PCA) and the determination of the probability of an individual being male by SVM analysis based on the abundance levels of the Vg isoforms (4, 7, 9) showed a clear separation of individuals into female and male clusters (Figure 1).

Differences between sexes on the molecular level were only obvious in animals sampled in the time period of reproduction (September ‐ January samplings). June is outside the reproductive time period of *E. verrucosus* and reproduction‐related physiological processes are therefore absent. Thus, it can be expected that expression levels of reproduction‐related female‐specific proteins, such as Vg, are likewise low in both female and male *E. verrucosus* in this month. The abundance levels of all Vg isoforms were low in the eleven *E. verrucous* individuals sampled in June (Figures A4 and A5A) and similar to those in male *E. verrucosus* sampled in the other months; however, it can be seen as highly unlikely that all individuals from June were males and female specimens were absent. Thus, in previous field samplings performed in June female *E. verrucosus* were found to be even more abundant than males: The female/male ratio was found to be about 2:1 [the sex of *E. verrucous* individuals was determined based on morphological sex features; (Bazikalova 1941; Govorukhina 2005); own field observations].

A PCA of the abundances of all annotated proteins of *E. verrucosus* from the field samplings showed clear, sex‐specific groupings of the proteomic data, with data for June samples in a separate cluster (Figure A5B, see also Figure 1). The first principal component explains over 14% of the variation in protein abundances; a cluster with the data for June samples is separate from the data for the samples from the other months, which do not show any further month‐specific grouping (Figure A5B). The second principal component separates female‐ and a male‐specific clusters with the data for the samples from September–January (Figure A5B). These groupings of the *E. verrucosus* proteomes are in agreement with the sex assignments based on the Vg levels used as molecular sex markers (Figures A4 and A5), supporting their validity. Notably, the data cluster for *E. verrucosus* sampled in June is spatially closer to the female than to the male data cluster for the individuals sampled in the other months (Figure A5B).

## Text S3. Sex‐Specific Abundance Levels of Proteins Showing Abundance Patterns Occuring in Both Sexes (Supplementary to Figures A2 and A3)

Certain proteins showed abundance changes in the proteomes of both female and male *E. verrucosus* from different samplings in the different months. Continuously increasing or decreasing trends of protein abundance levels were found for 41 and five proteins, respectively, over the fall to winter samplings (September to January; Figure A6A,B). The sex‐independent differential abundance trends correlated with the “overwintering” trait; therefore, it seems obvious they were related to proteome responses to the changing environmental conditions in the transition of seasons (fall to winter). The environmental parameters subjected to changes include water temperature (Table 1, see also Figure A1) and food abundance (Figure A2; see section “*Acclimation to season‐related environmental conditions*” in the Discussion). A clear trend of upregulation was seen for 28 other proteins in all examined individuals sampled in the summer (June; Figure A7). As female *E. verrucosus* are in an ovarian diapause during this month, the sex of the animals could not be determined. However, since this proteomic pattern was found in all examined animals that presumably included both sexes it can be assumed that the abundance patterns of those proteins occurred likewise in *E. verrucosus* of both sexes.

## Text S4. Highly Abundant Proteins (Supplementary for Results Sections “Female—Specific Proteome Characteristics” and “Male—Specific Proteome Characteristics”; Figure 4)

Proteins were defined as highly abundant when, in the proteome analysis with LC‐MS, median intensity was ≥ 2 × 10^6^, marking predominant proteins in the proteomes.

Proteins with high abundance levels in the proteomes can be used as indicators for primary cell‐physiological processes in the examined animals from a certain sampling.

In our study, highly abundant proteins in *E. verrucosus* were sex‐ and sampling month‐specific (Figure A8).

## Text S5. Upregulated Proteins Specific for Female *E. verrucosus* (Supplementary to Figures A4 and A5)

Proteins showing comparatively high abundance levels in the proteomes of female *E. verrucosus* were identified and used as indication for ongoing female‐specific physiological processes (see “*Female‐specific proteome characteristics*” in the Results section of the main paper).

The female *E. verrucosus* that could be examined were from the sampling months September to January and thus from within the time period of reproduction. Female and male *E. verrucosus* collected in the field in June could not be distinguished and thus female individuals were not identified as they were in a state of ovarian diapause (see Text S2).

Proteins that were differentially abundant in female *E. verrucosus* proteomes were assigned to modules (Figure 4). Sex‐ and sampling month‐ specific abundance levels of those proteins in the proteomes of *E. verrucosus* are shown as boxplots in Figure A9 (red module), Figure A10 (pink, magenta, greenyellow modules; ANGPTL4 as exceptionally highly expressed protein), and in Figure A11 (salmon module).

Differentially abundant proteins in female *E. verrucosus* can be associated to egg protein components [section (i) below] and to metabolism of egg components [sections (ii)—(iv) below]; to molting of the animal in November (v); to an increased oxygen demand in September (vi); and to other functions (vii).

### (i) Egg Protein Components

Using WGCNA of the here obtained proteomes, proteins were identified that all were upregulated in female *E. verrucosus* sampled in the fall‐winter months [September ‐ January samplings; Figure 4 (red module), Figure A9], including vitellogenins, clotting protein F12, pheromone/general odorant binding proteins, bifunctional purine biosynthesis protein ATIC, phosphoribosylformylglycinamidine synthase ade2, carboxypeptidase Q (Cpq), pantothenate kinase 2 (PANK2), and serine protease PRSS21. A likely source of those proteins, particularly for the major egg yolk protein vitellogenin (Hyne 2011), are the eggs located in the female gonads. Own unpublished data indicate that all of the listed proteins [except of one of two detected isoforms of pheromone/general odorant binding proteins (OBP)]—are egg‐specific. Those proteins showed increased abundance levels in the proteomes of female *E. verrucosus* from the fall/winter months but not of any individuals sampled in June when female *E. verrucosus* do not carry eggs (Figure A9).

We detected upregulation of five isoforms of vitellogenin (Vg), which is a member of the large lipid transfer protein family (LLTP) and a precursor of the yolk protein [Figure 4 (red module), Figures A4 and A9]. An occurence of diverse Vg isoforms was previously shown for the amphipod *G. fossarum* (Trapp et al. 2016); considering that various Vg isoforms were also identified for *E. verrucosus* in this study, it may indicate that diversification of Vg is common for amphipods.

Three isoforms of proteins related to pheromone/general odorant binding protein (OBP) were found to be upregulated in females sampled in the fall‐winter months [“pheromone/general odorant binding protein”, “uncharacterized protein LOC108673699”, “hypothetical protein FHG87_014485”; Figure 4 (red module), Figure A9]. The insect‐specific OBP are responsible for the detection of sex pheromones (Pelosi et al. 2018). Proteins from the pheromone/general odorant binding protein family were first described for Hexapoda species (Pelosi et al. 2018) and have more recently been studied in other athropods (Zhu et al. 2021). Proteins closely related to OBP from insects were found in amphipods from Lake Baikal (Drozdova et al. 2020). However, the functions of OBP of amphipods and of insects appear to differ. Amphipod OBP may be involved in carotenoid binding (Drozdova et al. 2020). The black color of the eggs of *E. verrucosus* in the first several months of development (Figure A3D) may be related to carotenoid pigment accumulation. This is commonly found for the eggs of many crustaceans and is supposed to play a vital role in antioxidant and UV radiation protection (de Carvalho and Caramujo 2017). Thus, the increased abundance of OBP in the proteomes of female *E. verrucosus* in the reproduction period may be related to the accumulation of carotenoids in the eggs carried by the *E. verrucosus* females.

The upregulated ATIC and ade2 proteins in this study are known to be involved in the purine biosynthetic pathway. Purine biosynthesis is essential in embryonic development in mammals (Alexiou and Leese 1992; Michot et al. 2017). The current study uncovered a similar need for purine biosynthesis enzymes in amphipods during reproduction.

Among proteins upregulated during the fall‐winter months, there were two proteases: carboxypeptidase Q (Cpq) and serine protease Prss21. The proteases in the eggs are known to degrade yolk protein to release nutrients during early embryo development in vertebrates, insects, and flatworms (Gerhartz et al. 1997; Indrasith et al. 1988; Mohamed et al. 2005). In crab embryos, proteases were shown to be involved in the initiation of hatching of juveniles from the egg (Devries and Forward 1991).

In the proteomes of *E. verrucosus* females from the samplings in the fall‐winter months pantothenate kinase 2 (PANK2) was upregulated (Figure 4, red module). PANK2 is involved in the first and rate‐determining step of coenzyme A (CoA) synthesis (Kotzbauer et al. 2005). Defects of the protein are associated with neurodegeneration in humans (Gregory and Hayflick 2005). In zebrafish embryos, pantothenate kinase was reported to be necessary for normal vascular development (Khatri et al. 2020).

### (ii) Proteins Involved in Production of Egg Lipoglycoproteins

Vitellogenesis, that is, the formation of yolk consisting of yolk proteins and lipids, is a key process during reproduction, typically occurring during precopulatory mate guarding (i.e., amplexus). Different enzyme types show high activity to produce high amounts of yolk lipoproteins. Vg maturation occurs in the endoplasmic reticulum and Golgi apparatus (Hinsch and Cone 1969). We observed upregulation of a group of proteins of the endoplasmic reticulum indicating an elevated oxygen demand in female *E. verrucosus* during precopulation in September and November (Figures 4, A10, pink module); it seems likely that this is related to the high rate of yolk lipoprotein synthesis. Moreover, the proteomes of females collected in September and November showed upregulation of two proteins involved in endoplasmic reticulum‐Golgi vesicle transport, COPB and USO1 (Figures 4, A10, pink module). This might be due to enhanced vesicle transport during Vg maturation. Furthermore, the abundance level of Gfat1 was increased in females sampled in November. Gfat1 is a rate‐limiting enzyme of the hexosamine pathway that is crucial for the production of a key substrate for protein glycosylation (Figures 4, A10, greenyellow module). As Vg undergoes glycosylation (Gottlieb and Wallace 1982; Tirumalai and Subramoniam 2001), upregulation of Gfat1 may be related to an increased need for a glycosylation substrate when the Vg synthesis for egg yolk is elevated.

### (iii) Potential Role of the Highly Abundant ANGPTL4 Protein in Lipid Enrichment in Eggs

The abundance levels of ANGPTL4 were among the highest in the proteomes of *E. verrucosus* females from amplexuses (Figures A8 and A10). This protein is known to increase the concentration of plasma triglycerides (TG) in vivo (Ge et al. 2004) through the inactivation of lipoprotein lipase LPL, a key enzyme in the decomposition of TG and phospholipids (PL) (Dugi et al. 1995; Jackson and Demel 1985). The lipid of the yolk in crustacean eggs almost entirely consists of TG and PL, serving as the primary energy source for the embryos (Graeve and Wehrtmann 2003). The high abundance of ANGPTL4 protein in precopulated females is likely related to the production of TG and PL, deposited in the eggs.

### (iv) Proteins Transporting Lipoprotein and Lipids

In female *E. verrucosus* from amplexuses a large subunit of microsomal triglyceride transfer protein (salmon module, Figure A11) and protein disulfide isomerase PDI (pink module, Figures 4 and A10A) were found to be upregulated. These two proteins compose a heterodimeric protein complex MTP, which assembles and secretes lipoproteins (Wetterau et al. 1990). Furthermore, MTP was observed to be involved in the reproduction of females in copepod *Lepeophtheirus salmonis*, in which it was hypothesized to transfer lipids from the intestine to growing oocytes (Khan et al. 2017).

In female proteomes from the November sampling, high abundance of the low‐density lipoprotein receptor LDLR was detected (greenyellow module Figures 4 and A10C). LDLR is known to be a receptor for lipoproteins, including Vg. Therefore, the abundance of LDLR in November can be assumed to relate to yolk protein incorporation into the oocyte, as it was reported for the prawn *Marsupenaeus japonicus* (Mekuchi et al. 2008).

### (v) Proteins Involved in the Molting Process

In the proteomes of female *E. verrucosus* sampled in November, increased abundances were found for Hexo1 and Cht5, responsible for chitin degradation, and for Cpap3 and verm, involved in the formation of a new cuticle (Figures 4 and A10B,C). These proteins may hence be related to molting (ecdysis) of female *E. verrucosus* during this period. Thus, female amphipods undergo molting prior to egg fertilization; it is assumed that it facilitates egg movement through the oviducts (Borowsky 1984).

A further candidate protein involved in molting is carboxylesterase, which regulates the molting cycle in the crustacean *Portunus trituberculatus* (Tao et al. 2017). Proteomes were specifically examined for an *E. verrucosus* homolog and, indeed, carboxylesterase Est‐6 abundance levels were found to be increased in females compared to males of *E. verrucosus* in amplexuses (September, November samplings; Figure A12).

Increased abundances of these proteins in November, close to the end of the precopulation phase, likely indicate that the female *E. verrucosus* were preparing molt prior to the fertilization of the eggs.

### (vi) Proteins Indicating Increased Oxygen Demand in Females in September

We found upregulation of several proteins indicating elevated oxygen demand in females in September: HYOU1 (Figures 4 and A10A), hemocyanin KLH1, and malate/L‐lactate dehydrogenase (Figure A11). Hemocyanin is a hemolymph transporter of oxygen in arthropods. The metabolic rate in reproducing females is high; their energy is primarily allocated to reproduction (Sokolova et al. 2012). The water temperature of 10.5°C at the sampling site in September was the highest of all sampling months (Table 1). This temperature is close to this upper thermal limit of *E. verrucosus* (Jakob et al. 2016). Females relocating energy to reproduction need more energy and oxygen to handle temperatures close to this upper thermal limit. In *Eulimnogammarus cyaneus* (Dybowsky, 1874), closely related to *E. verrucosus*, hemocyanin subunits were upregulated in females facing heat stress (Bedulina et al. 2017). Upon acute heat stress, both *E. verrucosus* and *E. cyaneus* exhibited upregulation of hemocyanin (Lipaeva et al. 2023). The high demand for oxygen in females in September is confirmed by the abundance of the hypoxia‐upregulated protein 1 (HYOU1) and malate/L‐lactate dehydrogenase, catalyzing the last step in anaerobic glycolysis.

### (vii) Proteins Related to Other Functions

The upregulation of metalloendoprotease neprilysin‐2 (Nep2) was detected in females from the November sampling (magenta module; Figures 4 and A10B). Neprilysin‐2 was shown to be crucial in *D. melanogaster* females for regular female reproductive fitness, particularly in sperm retention (Sitnik et al. 2014).

Spartin protein Spg20 was found to be specifically more highly abundant in the proteomes of female *E. verrucosus* sampled in November (greenyellow module; Figures 4 and A10C). Previous studies indicated that this protein can play a role in neurological disorders in humans; its function is related to regulating lipid droplet turnover (Eastman et al. 2009; Hooper et al. 2010). As studies on the function of the protein were in a neurological context, the background of its role in *E. verrucosus* remains unclear.

The abundance of the protein hemicentin HMCN1 was increased in the proteomes of females *E. verrucosus* sampled in amplexuses in September and November. Hemicentin was shown to be upregulated in queen bees after insemination and involved in visual perception (Liberti et al. 2019). In the silkworm *Bombyx mori*, hemicentin was detected as one of the members of the olfactory system network (Xin and Zhang 2021). Indeed, own research showed increased abundance of this protein in the antennae of female *E. verrucosus* (unpublished data). The antennae of amphipods function as an olfactory organ (Gentes and Scholtz 2019), and we thus assume that the increased abundance of HMCN1 could be related to a function in the olfactory perception of *E. verrucosus* females during the amplexus state. However, it remains unclear what its specific function might be and what has caused its increased abundance.

## Text S6. Abundances of Proteins Upregulated in Male *E. verrucous* During the Reproduction Period (SI for Section “Male‐Specific Proteome Characteristics” in the Results Section)

The abundance levels of proteins showing elevated abundances in males from amplexuses (September, November samplings) are depicted for both *E. verrucosus* sexes from all samplings in Figure A13.

The proteomes of the males did not show patterns that could clearly be linked to male‐specific reproduction‐related processes.

Males sampled in September and November showed upregulation of the proteins of the glycolysis pathway. This may be related to spermatogenesis; thus, upregulation of glycolytic proteins has been observed in the testes of *G. fossarum* during spermatogenesis (Esposti et al. 2019). Furthermore, a catalytic subunit of heterodimeric calcineurin PPP3CC was more highly abundant in males from theses sampling months than in females (Figure A13). The heterodimer of calcineurin (calcineurin A and B) is a calcium‐dependent serine/threonine protein phosphatase. Male reproductive organs of *Gammarus fossarum* were earlier found to be rich in calcium‐dependent proteins (Trapp et al. 2014), which was assumed to be due to sperm maturation, relying on the internal Ca^2+^ concentration. Furthermore, the isoforms of calcineurin A, PPP3CC, are known to compose testis‐enriched calcineurin and are involved in sperm motility in mammals (Liu et al. 2020; Miyata et al. 2015). Moreover, males from these sampling months showed upregulation of a group of proteins, which are structural components of the sarcomere, such as several isoforms of twitchin protein, titin (sls), and myozenin (Figure A13). In the amplexus state that can last for more than a month, males grasp female *E. verrucosus* with the dactyl under the females’ segments (see Figure A3B). We hypothesize that the observed upregulation of sarcomere proteins is related to the long‐term muscle activity when males grasp female *E. verrucosus*. Twitchin protein is well‐known for its involvement in a long‐term catch contraction in molluscan smooth muscle (Funabara et al. 2005). The arginine kinase (Argk), one of the major phosphagen kinases in crustaceans, was also upregulated in males in September and November (Figure A13). Argk activity was found to mainly occur in muscle tissue in arthropods (Abe et al. 2007; Walliman and Eppenberger 1973; Yao et al. 2009), with increases of protein abundance and activity under hypoxic conditions (Abe et al. 2007; Newsholme et al. 1978). The upregulation of Argk in *E. verrucosus* males from amplexuses indicates that their muscles utlize a high amount of energy, provided by ATP released from phosphagens. Another indication of increased energy consumption is the upregulation of 4‐alpha‐glucanotransferase (AGL) and glycogen phosphorylase (GlyP), which is involved in glycogen degradation.
